# Overcoming EGFR‐Mediated Dendritic Cell Dysfunction to Enhance Anti‐tumor Immunity in EGFR‐Mutant NSCLC by Precisely Targeting CD73 With pH‐responsive Nanocarriers

**DOI:** 10.1002/advs.202513182

**Published:** 2025-10-27

**Authors:** Xiaoling Shang, Xudong Geng, Zixu Wang, Shumin Yuan, Shanshan Ding, Ni Liu, Xinchun Ma, Xuan Sun, Huimin Wang, Ying Sun, Xun Qu, Guangwen Ren, Yong Qiang Li, Xiuwen Wang, Yanguo Liu

**Affiliations:** ^1^ Department of Medical Oncology Qilu Hospital of Shandong University 107 Wenhuaxi Road Jinan Shandong 250012 China; ^2^ The Jackson Laboratory Bar Harbor Maine 04609 USA; ^3^ Institute of Advanced Interdisciplinary Science School of Physics Shandong University Jinan Shandong 250100 China; ^4^ Department of Clinical Laboratory Shandong Cancer Hospital & Institute Shandong First Medical University & Shandong Academy of Medical Sciences Jinan Shandong 250117 China; ^5^ Department of Oncology Yantai Affiliated Hospital of Binzhou Medical University Yantai Shandong 264100 China; ^6^ Laboratory of Basic Medical Sciences Qilu Hospital of Shandong University 107 Wenhuaxi Road Jinan Shandong 250012 China; ^7^ Lung Cancer Center Qilu Hospital of Shandong University 107 Wenhuaxi Road Jinan Shandong 250012 China

**Keywords:** adenosine, dendritic cells, EGFR mutations, ^F127^ZIF‐8_AB680_, immune checkpoint inhibitors, non‐small cell lung cancer

## Abstract

EGFR mutations remain a major challenge in immunotherapy for non‐small cell lung cancer (NSCLC), with poor responses to immune checkpoint inhibitors driven by mechanisms associated with EGFR mutation‐mediated tumor microenvironment (TME) modulation. This study reveals that EGFR mutations prominently impaired dendritic cell (DC) maturation, disrupting their capacity to effectively prime CD8^+^ T cells and thereby compromising anti‐tumor immune responses. By application of clinical specimen analyses, multi‐omics approaches, and in vivo mouse models, this work demonstrates that EGFR mutations elicited adenosine production through the ERK/c‐Jun signaling axis in tumor cells, establishing an immunosuppressive TME that impeded maturation and antigen presentation of DCs, and in turn weakened CD8^+^ T cell activation. To overcome the EGFR mutation‐induced immunosuppression, this work next develops ^F127^ZIF‐8_AB680_, a pH‐responsive and tumor‐selective nanodrug specifically designed to target the CD73‐adenosine pathway within the acidic TME. This nanodrug significantly improves the therapeutic efficacy of PD‐1 blockade, leading to robust tumor growth inhibition and prolonged survival of mice in EGFR‐mutant NSCLC models. Leveraging the advanced nanotechnology, this newly designed pH‐sensitive nanocarrier introduces a precise CD73/adenosine inhibition within the acidic TME that reprograms the immune landscape in EGFR‐mutant NSCLC, which represents a promising therapeutic strategy to overcome immunotherapy resistance in NSCLC.

## Graphical abstract

EGFR mutations drive ERK/c‐Jun–mediated transcriptional activation of CD73, elevating adenosine levels and fostering an immunosuppressive TME by impairing DC maturation and CD8⁺ T cell priming. The pH‐responsive nanodrug ^F127^ZIF‐8_AB680_ selectively inhibits CD73, normalizes adenosine metabolism, and re‐establishes effective anti‐tumor immunity.

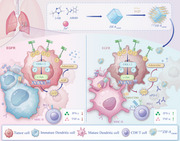

## 1 Introduction

Epidermal growth factor receptor (EGFR) mutations are prevalent in non‐small cell lung cancer (NSCLC), particularly in adenocarcinomas, and have reshaped the therapeutic landscape through the development of targeted therapies. ≈10–15% of lung adenocarcinoma cases in Caucasians and up to 50% in Asian populations harbor these mutations, with exon 19 deletions (19del) and the L858R point mutation in exon 21 being the most common variants.^[^ ^1^, ^2^ ^]^ Although these patients initially respond to targeted therapies, they usually become resistant to the therapy later. Thus, effective treatment strategies are urgently needed.^[^ ^3^ ^]^

The advent of immune checkpoint inhibitors (ICIs) targeting the PD‐1/PD‐L1 axis, which were designed to release the brakes on T cells, has demonstrated impressive anti‐tumor effects in advanced NSCLC, opening a new era in NSCLC treatment.^[^ ^4^, ^5^, ^6^, ^7^, ^8^ ^]^ Despite the promising results of ICIs in NSCLC, patients with EGFR‐mutant NSCLC demonstrated exceedingly poor clinical responses to anti‐PD‐1/PD‐L1 monoclonal antibodies (mAbs),^[^ ^9^, ^10^, ^11^, ^12^ ^]^ with some cases even progressing to hyper‐progressive disease, highlighting the imperative need to elucidate the mechanisms underlying this profound immune resistance. Existing research suggests that the immunosuppressive tumor microenvironment (TME) in EGFR‐mutant NSCLC plays a pivotal role in immune evasion. Contributing factors such as low infiltration of CD8^+^ tumor‐infiltrating lymphocytes (TILs), low tumor mutational burden (TMB), reduced neoantigen load, and increased presence of regulatory T cells (Tregs) and myeloid‐derived suppressor cells (MDSCs) have been reported.^[^ ^9^, ^13^, ^14^, ^15^ ^]^ However, these factors alone do not fully explain the compromised efficacy of ICIs in EGFR‐mutant tumors, suggesting that additional immune‐regulatory mechanisms may be at play.

While CD8^+^ T cells are central to anti‐tumor immunity, their effectiveness hinges on support from other immune cells within the TME. Studies suggest that solely reversing T cell inhibition does not necessarily enhance therapeutic outcomes, as a functional immune response requires the coordination of multiple types of immune cells.^[^ ^16^ ^]^ Among these, dendritic cells (DCs) are indispensable for priming CD8^+^ T cells by capturing and presenting tumor antigens, a process that initiates and sustains the adaptive immune response.^[^ ^17^, ^18^, ^19^ ^]^ As the primary link between the innate and adaptive immunity, DCs are uniquely equipped to orchestrate anti‐tumor responses by activating naïve T cells, providing co‐stimulatory signals, and regulating the balance between immunity and tolerance. Tumor‐associated DCs, however, often display an immature phenotype, frequently exhibiting functional deficits and limited immune‐stimulatory capacity, which cause significantly weakened anti‐tumor responses. These dysfunctional DCs not only fail to effectively activate T cells but may also contribute to immune tolerance or immunosuppression by secreting immunosuppressive cytokines or promoting the expansion of Tregs. Although recent studies have indicated that EGFR mutations may reshape the immune landscape in NSCLC, their specific impact on DC functionality within the TME has not been well characterized. Understanding the mechanisms by which EGFR mutations disrupt DC function is essential for identifying therapeutic targets that could improve the efficacy of immunotherapies in this population of patients.

In the present study, adenosine, a purine nucleoside that accumulates in the TME and exerts potent immunosuppressive effects, was identified as one prominent factor contributing to the immune evasion of EGFR‐mutant tumors. Previous studies have demonstrated that elevated levels of adenosine in the TME suppresses the activity of immune effector cells, including T cells, DCs, NK cells and macrophages, further promoting an immunosuppressive environment.^[^ ^20^, ^21^, ^22^, ^23^ ^]^ Inhibition of CD73, the primary enzyme responsible for adenosine production, could represent a promising strategy to counteract adenosine‐associated immune suppression and enhance the efficacy of ICIs in EGFR‐mutant NSCLC. However, current small‐molecule inhibitors targeting adenosine, including CD73 inhibitors, showed limited efficacy in clinical trials.^[^ ^24^, ^25^ ^]^ These limitations stem from several critical shortcomings of the drugs, such as their poor stability in biological systems, their suboptimal bioavailability, and rapid systemic clearance, which collectively hinder their therapeutic potential. More importantly, these inhibitors may fail to achieve effective accumulation at tumor sites, limiting their ability to adequately modulate the immunosuppressive TME.^[^ ^26^ ^]^ Thus, while promising in concept, the CD73 inhibitors often lack tumor‐specific delivery mechanisms, reducing their impact on the immunosuppressive niches created by EGFR‐mutant tumors. Altogether, the inefficacy of current adenosine‐targeting approaches highlights a significant therapeutic gap in leveraging small‐molecule inhibitors to remodel the adenosine‐associated immunosuppression within the TME and addressing these challenges requires innovative approaches to overcome above‐mentioned pharmacokinetic and pharmacodynamic limitations.

To this end, we developed ^F127^ZIF‐8_AB680_, a pH‐responsive nanoparticle that selectively inhibits CD73 and reduces adenosine production, and more importantly, precisely delivers the drug into the acidic TME. By restoring the DC maturation and enhancing their capacity to prime CD8^+^ T cells, this approach reprograms the TME of EGFR‐mutant NSCLC, fostering more effective anti‐tumor immune responses than the traditional drug delivery method. Further, ^F127^ZIF‐8_AB680_ significantly enhanced the therapeutic efficacy of anti‐PD‐1 in mouse models, which represents a promising strategy for improving the therapeutic outcomes of EGFR‐mutant NSCLC patients suffering from immunotherapy resistance.

## 2 Results

### 2.1 Enriched Immunosuppressive DCs in EGFR‐Mutant NSCLC Tumors

**Figure 1:**
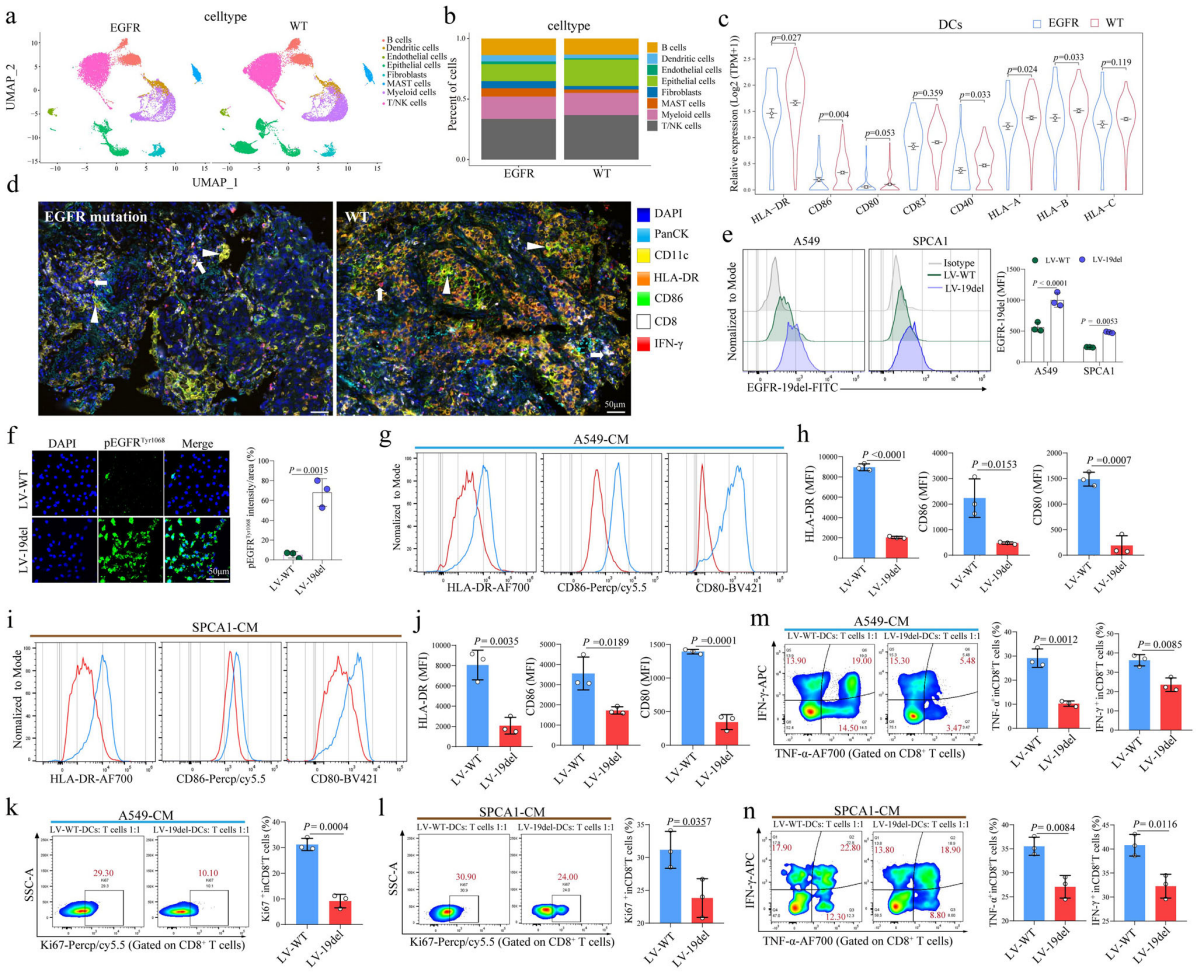
Immune Landscape and DC Maturation in EGFR‐Mutant NSCLC. a) UMAP plot of scRNA‐seq data illustrating the distribution of various immune cell types in the TME of EGFR‐mutant and EGFR WT NSCLC patients. b) Bar plot showing the comparative proportions of different immune cell populations between EGFR‐mutant and WT patients. c) Violin plots depicting the distribution of DC maturation markers expression levels in EGFR‐mutant versus EGFR WT NSCLC patients. d) Representative images of mIF images of FFPE tumor sections showing the infiltration of CD11c^+^HLA‐DR^+^, CD11c^+^CD86^+^ DCs and CD8^+^ lymphocytes in tumor tissues from EGFR‐mutant and WT patients. Markers for PanCK (light blue), CD11c (in yellow), HLA‐DR (orange), CD86 (green), CD8 (white) and IFN‐γ (red). Scale bar, 50µm. e) Fluorescence intensity of transfected A549 and SPCA‐1 cells, demonstrating successful transfection of the EGFR exon 19 deletion virus. f) Fluorescence‐based detection of EGFR phosphorylation (Tyr1068) in EGFR‐mutant and WT transfected cells. g‐j) Flow cytometry plots showing reduced expression of HLA‐DR, CD86, and CD80 on DCs cultured with supernatants from EGFR‐mutant versus WT cells. k–n) Proliferation (Ki67) and cytokine secretion (IFN‐γ and TNF‐α) in CD8^+^ T cells co‐cultured with DCs exposed to EGFR‐mutant versus WT conditioned media.

To dissect whether there is a unique immune cell composition within the TME of advanced EGFR‐mutant NSCLC, single‐cell RNA sequencing (scRNA‐seq) data from the GSE131907 dataset^[^ ^27^ ^]^ were analyzed (**Figure** **1**a,b). Notably, in the EGFR‐mutant group, there are increased ratios of DCs, fibroblasts, and mast cells, but reduced T and NK cells, in comparison to the EGFR wild‐type (WT) group. Further analysis on the functional DC subgroups revealed that EGFR‐mutant patients had significantly lower expression of the key maturation markers, including human leukocyte antigen‐DR (HLA‐DR) (*p* = 0.027), CD86 (*p* = 0.004), and CD40 (*p* = 0.033), with a marginal reduction in CD80 (*p* = 0.053), compared to the EGFR WT patients (Figure 1c).

Via Tumor Immune Estimation Resource (TIMER),^[^ ^28^ ^]^ we next determined whether the EGFR mutation may influence the abundance of immune infiltrates (B cells, CD8^+^ T cells, CD4^+^ T cells, macrophages, neutrophils, and DCs). Clearly, a higher degree of DCs (*p* < 0.01) and B cells (*p* < 0.05) were identified to be infiltrated in EGFR‐mutant tumors (Figure S1a, Supporting Information), and specifically, immature DCs (iDCs) showed a marked increase in the EGFR‐mutant TME, compared to the WT (*p* < 0.05) (Figure S1b, Supporting Information). These findings suggest that although there is an elevated infiltration of DCs in EGFR‐mutant tumors, the majority of them remain immature, which may potentially impair their antigen‐presenting functions and subsequently limit their effective activation of the adaptive immunity.

Furthermore, we examined the phenotype of DCs in the peripheral blood (PB) of 20 advanced NSCLC patients treated at our hospital, comprising 9 patients with EGFR mutation and 11 patients with WT. Flow cytometry analysis revealed no significant difference in the percentage of CD11c^+^Lin(CD3, CD14, CD19, CD20, CD56)^−^ cells between the EGFR mutation and WT groups (Figure S1c, Supporting Information). Among the functional and maturation markers examined, however, we observed a significant reduction in the expression of HLA‐DR (*p* < 0.0001) and CD86 (*p* = 0.0032) on DCs in the PB of patients with EGFR mutation compared to those with WT (Figure S1d–g, Supporting Information). Supporting this finding, multiplex immunofluorescence (mIF) staining of the formalin‐fixed paraffin‐embedded (FFPE) tissues collected from NSCLC patients showed a significant decrease in tumor‐infiltrating CD11c^+^HLA‐DR^+^, CD11c^+^CD86^+^cells and CD8^+^ lymphocytes cells in EGFR mutant patients compared to the WT (Figure 1d and Figure S2, Supporting Information). Collectively, these results strongly indicated that EGFR mutation reshapes the phenotype of DCs toward an immature phenotype in both the TME and circulation, which may potentially impair anti‐tumor immunity.

### 2.2 EGFR‐Mutant Tumor Cells Drives DCs to Lose Their Ability to Prime CD8⁺ T Cells

To characterize the association between EGFR mutation and DC functions, we next constructed EGFR‐mutant human lung cancer cell lines by transfecting the EGFR exon 19del‐expressing virus into A549 or SPCA‐1 cells (Figure 1e). In the successfully constructed EGFR‐19del tumor cells, their phosphorylation levels of EGFR were notably elevated compared to the EGFR‐WT cells (*p* = 0.0015), indicating an enhanced activation of the EGFR signaling (Figure 1f). To test the possibility that the EGFR‐mutant tumor cells‐derived products could directly modulate DCs, we co‐cultured iDCs from healthy human blood monocytes with the conditioned medium (CM) collected from EGFR‐mutant or WT lung cancer cells and subsequently induced them to be mature with LPS (Figure S3a, Supporting Information). Remarkably, the EGFR mutant tumor cells products reduced the expression of HLA‐DR, CD86, and CD80 on DCs compared to WT cells (all *p* < 0.05) (Figure 1g–j, Figure S3b, Supporting Information), although they did not significantly alter the phagocytic ability of DCs (Figure S3c–g, Supporting Information). As a result, DCs exposed to CM from EGFR‐mutant tumor cells displayed diminished capacity to prime human CD8⁺ T cells, as shown their significant reduced proliferation and cytokine secretion (Figure 1k–n). Thus, EGFR‐mutant tumors impair DC maturation, leading to their inability to activate CD8⁺ T cells which may contribute to immune evasion.

**Figure 2:**
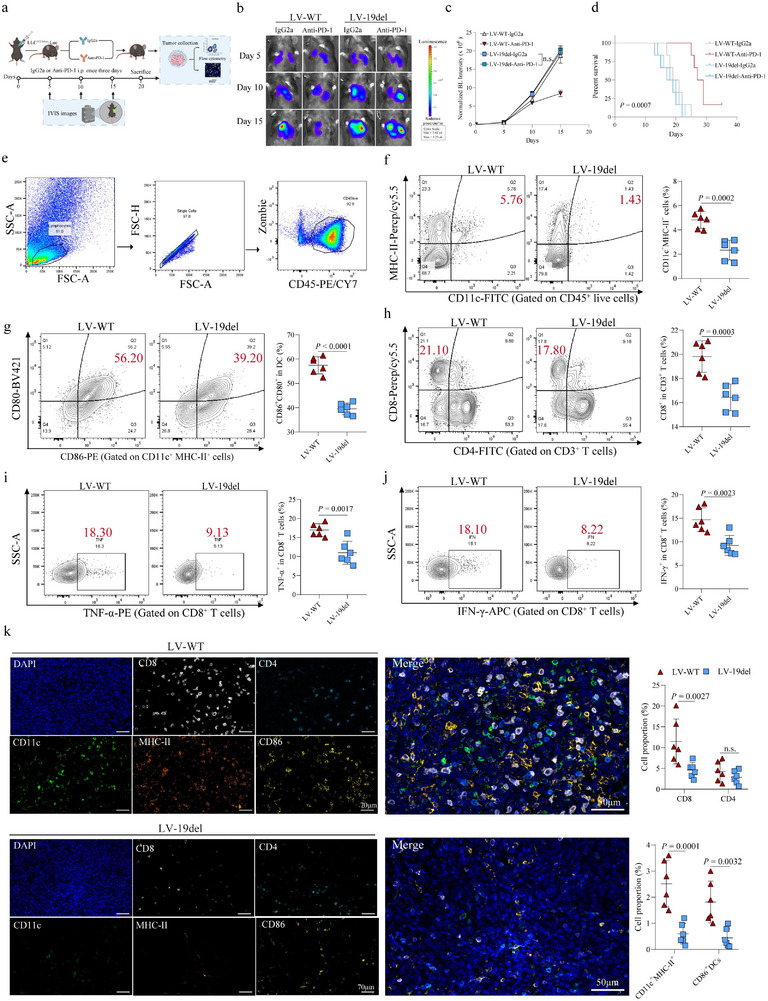
Treatment Effects and Immune Cell Infiltration in EGFR‐19del and WT Tumor‐Bearing Mice. a) Schematic outlining the experimental setup and treatment groups with Anti‐PD‐1 or IgG2a administration in mice bearing EGFR‐19del or WT LLC tumors. b) Representative bioluminescence images of tumor progression at days 5, 10, and 15 post‐tumor implantation. c) Tumor size quantification over time based on bioluminescence intensity across different treatment groups. d) Kaplan‐Meier survival curves comparing overall survival among the treatment groups. e) Gating strategy for flow cytometric analysis of immune cell populations in tumor tissues. f) Flow cytometry analysis of CD11c^+^MHC‐II^+^ DC infiltration in tumor tissues from EGFR‐19del and WT tumor‐bearing mice. g) Proportion of mature DCs (CD86^+^CD80^+^) in tumor tissues assessed by flow cytometry. h) Percentage of CD8^+^ T cells infiltration in the TME. i) Proportion of CD8^+^TNF‐α^+^ T cells in the tumor, indicating cytotoxic T cell activity. j) Percentage of CD8^+^IFN‐γ^+^ T cells within the TME. k) mIF staining showing CD8^+^ (white), CD4^+^ (light blue), CD11c^+^ (green), MHC‐II^+^ (orange), and CD86^+^ (yellow) immune cell distribution in tumor tissues from different treatment groups, Scale bar, up panel: 70µm; down panel: 50µm. (n = 6 samples per group).

To validate the above in vitro findings from human cells, we next constructed murine lung cancer line LLC cells expressing human EGFR‐19del (Figure S4a, Supporting Information) for the following in vivo models. As expected, compared to EGFR WT cells, EGFR19del‐transfected LLC cells showed increased sensitivity to gefitinib (EGFR inhibitor) treatment, indicating the successful establishment of this model (Figure S4b, Supporting Information). In an orthotopic model that EGFR‐19del or EGFR‐WT LLC cells were transplanted into the syngeneic C57BL/6J mice via intrathoracic injection (Figure S4c,d, Supporting Information), we then assessed how EGFR‐mutated tumors responded to immune checkpoint blockade anti‐PD‐1 (**Figure** **2**a). Mutation of EGFR rendered the tumors completely non‐responsive to anti‐PD‐1 treatment (Figure 2b–d). Consistent with the tumor responses, EGFR‐19del LLC tumors contain significantly lower numbers of CD11c^+^MHC‐II^+^ DCs (Figure 2e,f), which express reduced levels of CD80 and CD86, than the WT tumors (Figure 2g). Corresponding to the dysfunctional DCs, the total number of CD8^+^ T cells, as well as the CD8^+^TNF‐α^+^and CD8^+^IFN‐γ^+^ cytotoxic T lymphocytes (CTLs), were all significantly decreased in the TME of EGFR‐19del LLC tumors (Figure 2h–j). Supporting the flow cytometry results, the EGFR mutation‐associated DC dysfunction was also revealed by mIF staining (Figure 2k). Taken together, our in vitro and in vivo results indicated that EGFR‐mutant tumors inhibit DC recruitment and maturation, thereby impairing the activation of adaptive immune responses.

### 2.3 EGFR‐Mutant Tumor Cells Produce Elevated Level of Adenosine

**Figure 3:**
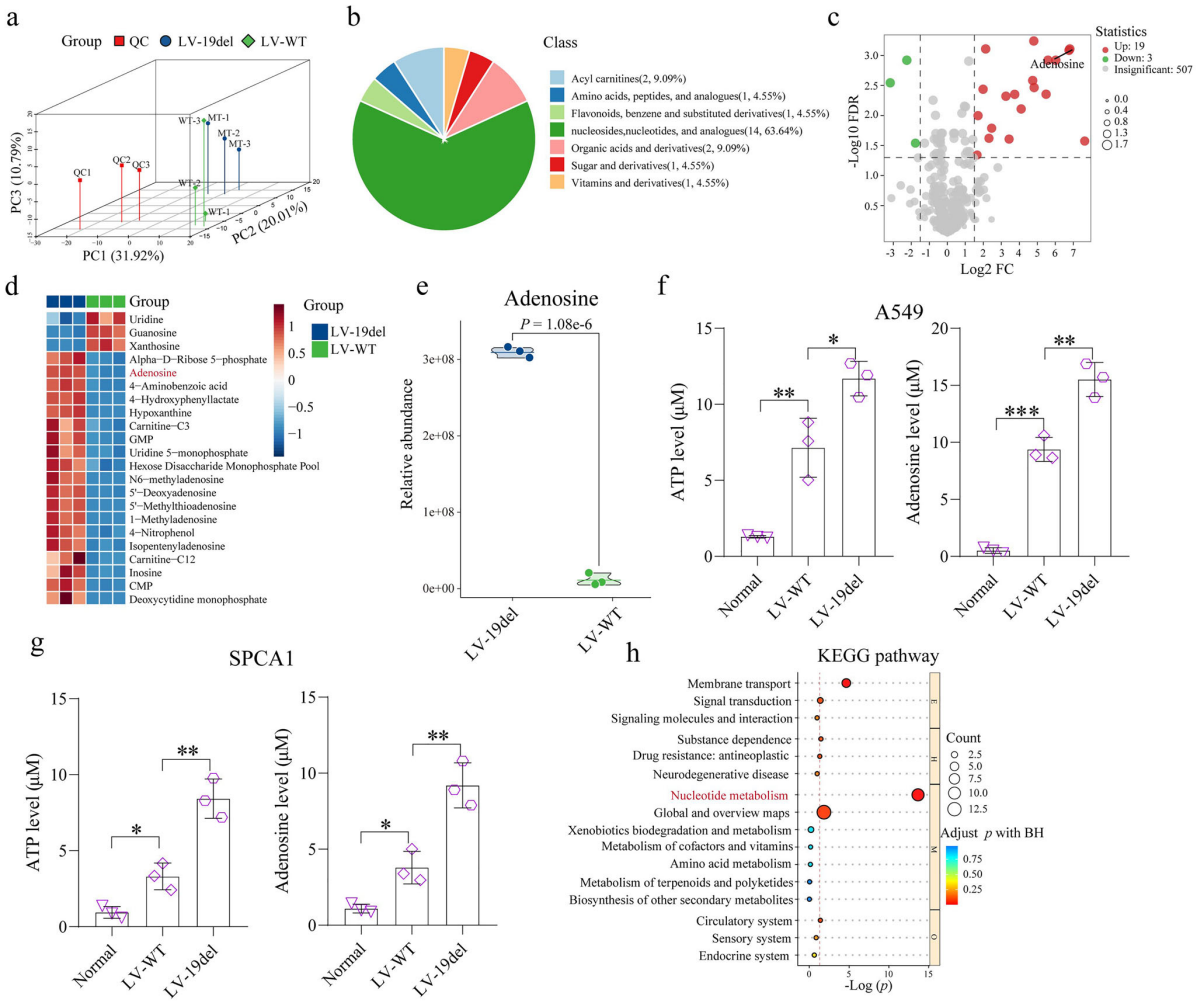
Metabolomic Profiling and Key Metabolic Pathway Alterations in EGFR‐WT and EGFR‐19del Cells. a) Principal component analysis (PCA) of metabolomic data from cell culture supernatants, showing separation between EGFR‐19del and WT groups. b) LC‐MS‐based identification of seven distinct metabolites between EGFR‐19del and WT cells. c) Volcano plot of differential metabolites in cell supernatants comparing EGFR‐19del and WT cells. d) Heatmap illustrating the clustering of differential metabolites across both groups. e) Violin plot comparing adenosine levels across both groups. f) Quantification of ATP and adenosine levels in A549‐19del, A549‐WT cells and BEAS‐2B (Normal) using ATP and adenosine detection assays. g) Quantification of ATP and adenosine levels in SPCA1‐19del, SPCA1‐WT cells and BEAS‐2B (Normal) using detection kits. h) KEGG pathway enrichment analysis highlighting altered metabolic pathways, focusing on nucleotide metabolism. *p < 0.05, **p < 0.01, ***p < 0.001.

Tumor metabolism plays a critical role in immune evasion through the generation of immunosuppressive metabolites. To investigate the mechanism by which EGFR mutations cause DC dysfunction, we conducted liquid chromatography‐mass spectrometry (LC‐MS) analysis of CM from A549 EGFR‐19del versus WT cells. The principal component analysis (PCA) score plot revealed a clear separation between the EGFR‐19del and EGFR‐WT groups due to biological differences (**Figure** **3**a), with seven distinct groups of metabolites identified (Figure 3b). Then, we applied a filtering process based on Student's t test (FDR‐corrected *p* < 0.05), the variable importance in projection (VIP) technique (VIP >1) and the fold‐change (|Log2FC| > 1.5) to screen for the differential metabolites between two groups, and 19 upregulated and 3 downregulated metabolites were subsequently identified (Figure 3c,d). Notably, adenosine, a known immunosuppressive metabolite,^[^ ^29^ ^]^ was found markedly upregulated in the EGFR‐19del CM (Figure 3c–e). Using adenosine detection kits, we indeed detected significantly higher levels of ATP and adenosine produced by EGFR‐mutant cells (A549‐19del and SPCA1‐19del) than EGFR‐WT cells (Figure 3f,g). In parallel, normal lung epithelial cells were included as a negative control, and adenosine production in these cells was far lower than that in tumor cells, further supporting that the elevated adenosine levels are tumor cell‐specific. To further clarify the cellular sources of adenosine in the TME, we dissociated orthotopic lung tumors into single cells and sorted tumor cells, fibroblasts, and Tregs by flow cytometry. Adenosine detection assays revealed that the majority of adenosine was produced by tumor cells, particularly in EGFR‐mutant tumors, whereas fibroblasts and Tregs contributed minimally (Figure S5a,b, Supporting Information). Further KEGG pathway enrichment analysis of the LC‐MS results revealed profound disruptions of the nucleotide metabolism pathway (Figure 3h), implying the adenosine's potentially immunosuppressive role in the TME. We then speculated that the increased adenosine may contribute to DC dysfunction and derailed CD8⁺ T cell activation characterized in the EGFR‐mutant TME.

### 2.4 CD73 is Overexpressed in EGFR‐Mutant NSCLC and Correlates with Poor Prognosis

**Figure 4:**
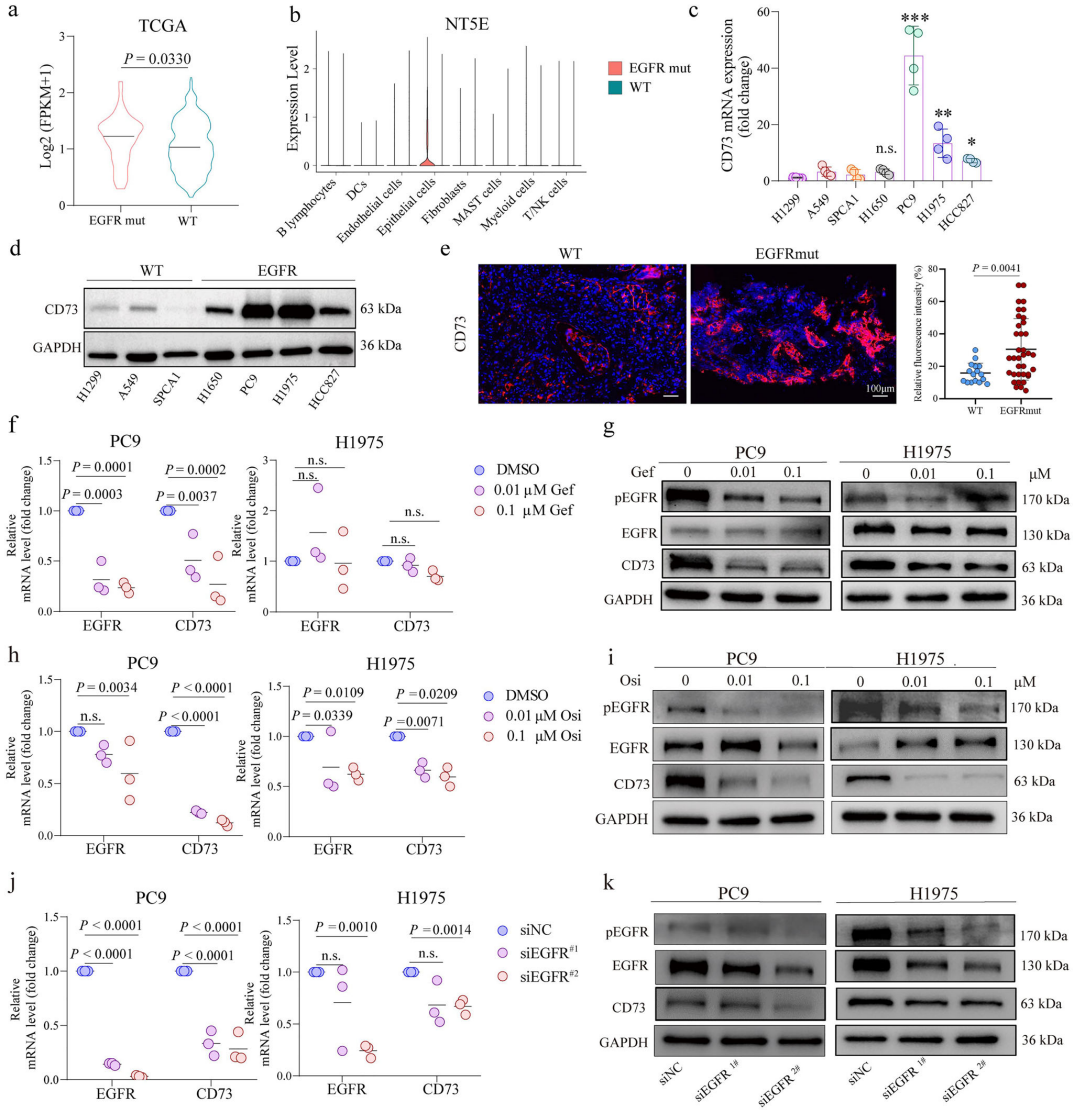
CD73 Expression is Regulated by EGFR in NSCLC. a) TCGA dataset analysis comparing CD73 (NT5E) mRNA expression levels between EGFR‐mutant and WT NSCLC patients. b) Violin plots from scRNA‐seq data showing CD73 expression levels across various cell types, with a focus on malignant epithelial cells in EGFR‐mutant tumors. c) RT‐qPCR analysis comparing CD73 mRNA expression in WT (H1299, A549, SPCA1) and EGFR‐mutant (H1650, PC9, H1975, HCC827) lung cancer cell lines. d) Immunoblotting analysis assessing CD73 protein expression levels across the same WT and EGFR‐mutant cell lines as in panel c. e) Representative images and quantification of Immunofluorescence staining showing CD73 expression in FFPE tumor tissues from 36 EGFR‐mutant and 16 WT NSCLC patients. Scale bar: 100µm. f) RT‐qPCR analysis of CD73 and EGFR mRNA expression following treatment with increasing concentrations of gefitinib in PC9 and H1975 cells. g) Immunoblotting blot analysis of CD73, EGFR, and phosphorylated EGFR protein levels in PC9 and H1975 cells treated with gefitinib. h) RT‐qPCR analysis of CD73 and EGFR mRNA expression following osimertinib treatment in PC9 and H1975 cells. i) Immunoblotting analysis of CD73, EGFR, and phosphorylated EGFR protein levels in PC9 and H1975 cells treated with osimertinib. j) RT‐qPCR analysis of CD73 and EGFR mRNA expression following EGFR knockdown in PC9 and H1975 cells. k) Immunoblotting analysis of CD73, EGFR, and phosphorylated EGFR protein levels following EGFR knockdown in PC9 and H1975 cells.

CD73, a key enzyme governing adenosine production, has been reported to regulate cancer progression by suppressing the anti‐tumor immunity within the TME.^[^ ^30^ ^]^ In TCGA dataset, the expression of NT5E, the CD73‐encoding gene, was detected to be significantly higher in EGFR‐mutant NSCLC tumors than that in the WT group (*p* = 0.0330) (**Figure** **4**a). Corroborating to this finding, analysis of an NSCLC scRNA‐seq dataset (GSE131907)^[^ ^27^ ^]^ revealed that the NT5E expression was predominantly contributed by malignant epithelial cells, with particularly higher levels in EGFR‐mutant tumors than the WT (Figure 4b). In human NSCLC cell lines, including 3 EGFR‐WT and 4 EGFR‐mutant lines, there was a significantly elevated CD73 expression at both mRNA and protein levels in EGFR‐mutant cells (Figure 4c,d). Supporting the above dataset and cell line results, immunofluorescence staining of the FFPE tumor tissues from EGFR‐mutant (n = 36) and EGFR‐WT (n = 16) NSCLC patients from Shandong Cancer Hospital also showed profoundly increased CD73 protein expression in EGFR‐mutant tissues (Figure 4e). Moreover, among these patients, survival analysis of 24 EGFR‐mutant NSCLC patients revealed that those with high CD73 expression had significantly worse overall survival (OS) than those with low expression (Figure S6a, Supporting Information). Meanwhile, multivariate Cox analysis further identified low CD73 expression as an independent prognostic factor for improved survival in EGFR‐mutant patients (HR = 0.198, 95%CI: 0.054–0.730; *p* = 0.015) (Figure S6b, Supporting Information).

### 2.5 Activated ERK Signaling Mediated EGFR‐driven CD73 Expression in Tumor Cells

To elucidate the association between EGFR activation and CD73 expression, we treated EGFR‐mutant PC9 and H1975 cells with the first‐generation EGFR tyrosine kinase inhibitor (TKI) gefitinib and third‐generation TKI osimertinib. While gefitinib decreased CD73 expression at both mRNA and protein levels in PC9 cells but not in gefitinib‐resistant H1975 cells (Figure 4f,g, Figure S6c,d, Supporting Information), osimertinib was able to downregulate CD73 expression in both H1975 and PC9 cells in a dose‐dependent manner (Figure 4h,i, Figure S6e,f, Supporting Information). Consistently, silencing EGFR with siRNA in both PC9 and H1975 cells led to reduced CD73 expression at both the mRNA and protein levels (Figure 4j,k, Figure S6g,h, Supporting Information). On the other hand, stimulation of human NSCLC cells with exogenous EGF (Figure S7a,b, Supporting Information) or transfecting the cells with EGFR‐overexpressing plasmids (Figure S7c,d, Supporting Information) or the EGFR‐19del virus (Figure S7e,f, Supporting Information) enhanced the EGFR phosphorylation (Tyr1068) and CD73 expression. Therefore, EGFR activation directly induces CD73 expression, suggesting a functional role for CD73 in EGFR‐mutant NSCLC.

To further validate these findings in *vivo*, we established an orthotopic lung tumor model with EGFR‐19del LLC cells. Saline (Ctrl) or Osimertinib was administered by oral gavage at a dose of 2 mg kg^−1^ qd. Tumor growth was assessed at the start of treatment, and after 2 and 3 weeks of treatment using bioluminescence imaging. Osimertinib treatment markedly inhibited tumor growth compared with vehicle control, as evidenced by representative bioluminescent imaging and tumor growth curves (Figure S8a,b, Supporting Information). In parallel, osimertinib significantly reduced CD73 expression and adenosine levels within the TME (Figure S8c,d, Supporting Information). Notably, osimertinib increased the abundance of tumor‐infiltrating DCs and, more importantly, enhanced their maturation capacity, indicating a partial restoration of DC function (Figure S8e–g, Supporting Information). Collectively, these clinical and in *vivo* findings demonstrate that CD73 is markedly upregulated in EGFR‐mutant NSCLC, and this aberrant expression not only predicts poor prognosis but also contributes to immunosuppressive remodeling of the TME.

**Figure 5:**
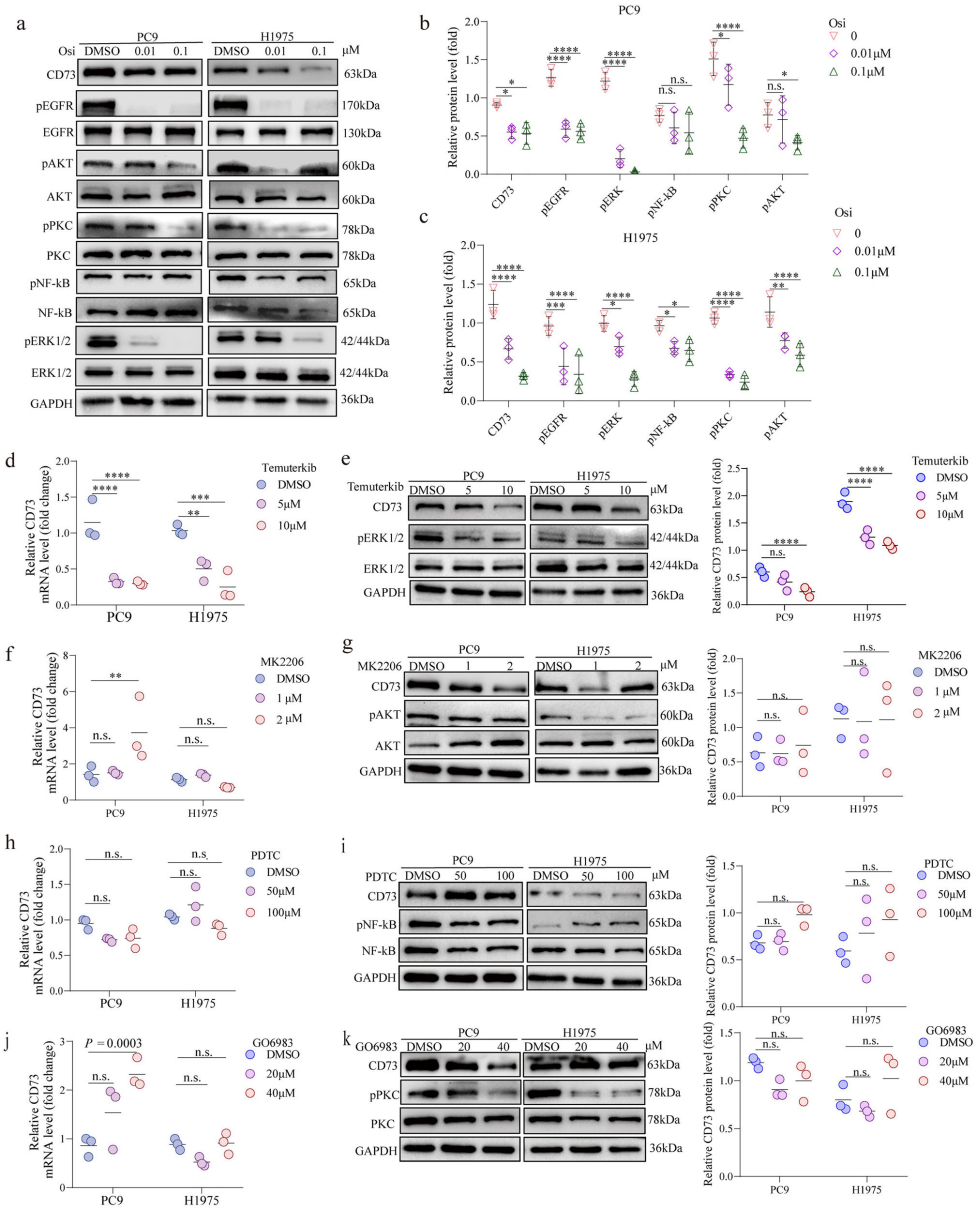
ERK Signaling Pathway Regulates CD73 Expression in EGFR‐Mutant Cells. a–c) Immunoblot and quantification analysis of PC9 and H1975 cells treated with osimertinib, showing the phosphorylation status of key EGFR downstream signaling molecules (ERK, AKT, NF‐kB, PKC). d) RT‐qPCR analysis of CD73 mRNA levels following treatment with the ERK inhibitor, Temuterkib, in PC9 and H1975 cells. e) Immunoblot and quantification analysis of CD73, total ERK, and phosphorylated ERK in PC9 and H1975 cells treated with Temuterkib. f,g) Effects of the AKT inhibitor, MK2206, on CD73 mRNA (f) and protein (g) expression in PC9 and H1975 cells, analyzed by RT‐qPCR and immunoblotting. h,i) Analysis of CD73 expression after treatment with the NF‐kB inhibitor, PTDC, using RT‐qPCR (h) and immunoblotting (i) in PC9 and H1975 cells. j,k) CD73 expression following PKC inhibition by GO6983, analyzed by RT‐qPCR (j) and immunoblotting (k), revealing the limited impact of PKC signaling on CD73 levels. *p < 0.05, **p < 0.01, **p < 0.001, ****p < 0.0001, n.s., no significance.

To characterize the molecular mechanism underlying EGFR‐driven CD73 expression, we examined the key reported signaling pathways downstream of EGFR, including ERK, PKC, NF‐κB, and AKT.^[^ ^31^ ^]^ Inhibition of EGFR with osimertinib resulted in reduced phosphorylation of ERK, NF‐κB, PKC, and AKT in both PC9 and H1975 cells, suggesting activation of all of these pathways downstream of EGFR (**Figure** **5**a–c). RT‐qPCR and immunoblotting analyses demonstrated that treatment with the ERK inhibitor significantly decreased CD73 mRNA and protein expression in both PC9 and H1975 cells (Figure 5d,e), indicating that ERK activity is essential for maintaining CD73 levels downstream of EGFR. In contrast, inhibition of AKT (Figure 5f,g), NF‐κB (Figure 5h,i), or PKC (Figure 5j,k) pathways had little to no effect on CD73 expression, suggesting that these pathways may not play a major role in mediating EGFR‐driven CD73 upregulation. These findings indicated that EGFR‐driven CD73 expression is mediated through the ERK signaling pathway in human NSCLC cells.

### 2.6 c‐Jun Regulates CD73 Expression by Binding to The CD73 Promoter

Using the JASPAR database,^[^ ^32^ ^]^ which contains curated, non‐redundant transcription factor (TF) binding profiles, we identified c‐Jun as a potential transcription factor downstream of the ERK signaling pathway that could regulate CD73 expression (data not shown). Indeed, upon exogenous EGF stimulation or overexpression of EGFR or EGFR‐19del in EGFR^WT^ A549 and SPCA1, c‐Jun expression was remarkably upregulated (Figure S9a–c, Supporting Information). Conversely, inhibition of EGFR with gefitinib decreased c‐Jun expression in EGFR^mut^ PC9 cells but not in the gefitinib‐resistant H1975 cells (Figure S9d, Supporting Information), while osimertinib downregulated c‐Jun levels in both cell lines in a dose‐dependent manner (Figure S9e, Supporting Information). Consistently, the knockdown of EGFR similarly led to reduced c‐Jun expression in PC9 and H1975 cells (Figure S9f, Supporting Information). Thus, c‐Jun is likely a key transcriptional factor controlling EGFR‐mediated CD73 expression in EGFR mutant tumor cells.

**Figure 6:**
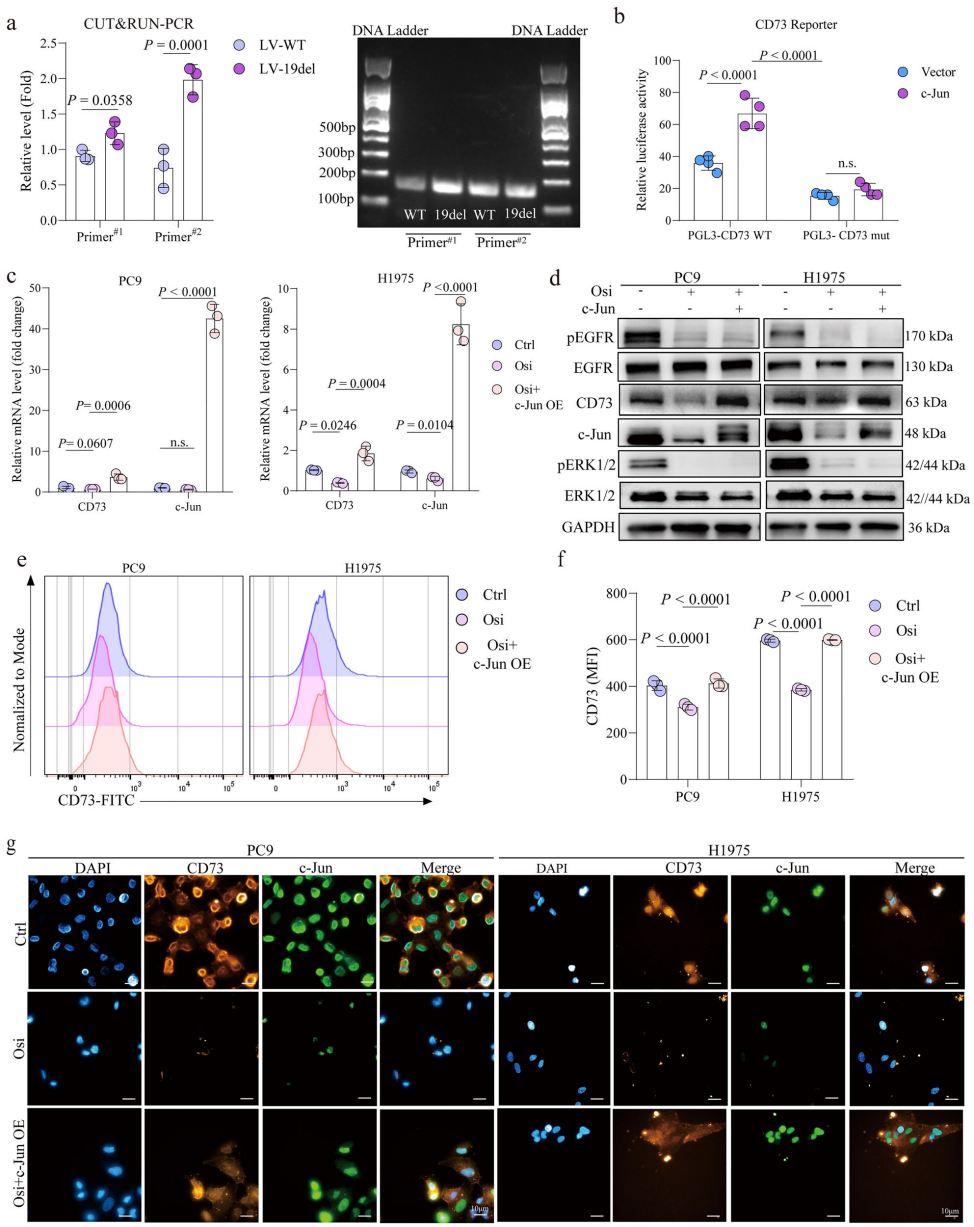
c‐Jun Mediated Regulation of CD73 Expression in EGFR mutant NSCLC cells. a) CUT&RUN‐qPCR analysis showing the binding of c‐Jun to the CD73 promoter (left). PCR product size verification via gel electrophoresis (right). b) Dual‐luciferase reporter assay assessing c‐Jun's interaction with the CD73 promoter. c) RT‐qPCR analysis of c‐Jun and CD73 expression in PC9 and H1975 cells treated with osimertinib alone or osimertinib combined with c‐Jun overexpression. d) Immunoblotting analysis of EGFR, p‐EGFR, ERK1/2, p‐ERK1/2, c‐Jun, and CD73 protein levels in PC9 and H1975 cells after treatment with osimertinib or osimertinib plus c‐Jun overexpression. e) Flow cytometry analysis of CD73 expression in PC9 and H1975 cells treated as described. f) Quantification of mean fluorescence intensity (MFI) of CD73 from flow cytometry data. g) Immunofluorescence staining of c‐Jun and CD73 in PC9 and H1975 cells following treatment with osimertinib or osimertinib combined with c‐Jun overexpression.

Supporting this notion, a CUT&RUN‐qPCR assay revealed that c‐Jun directly binds to the CD73 promoter, regulating its transcription (**Figure** **6**a). A further dual‐luciferase reporter assay validated this finding (Figure 6b). In rescue experiments, osimertinib‐induced downregulation of CD73 at both mRNA (Figure 6c) and protein levels (Figure 6d–g, Figure S10a,b, Supporting Information) was reversed by c‐Jun overexpression in EGFR^mut^ PC9 and H1975 cells. These results highlighted that c‐Jun plays an essential role in regulating CD73 expression.

### 2.7 Synthesis and Characterization of pH‐responsive Nanocarrier ^F127^ZIF‐8_AB680_

**Figure 7:**
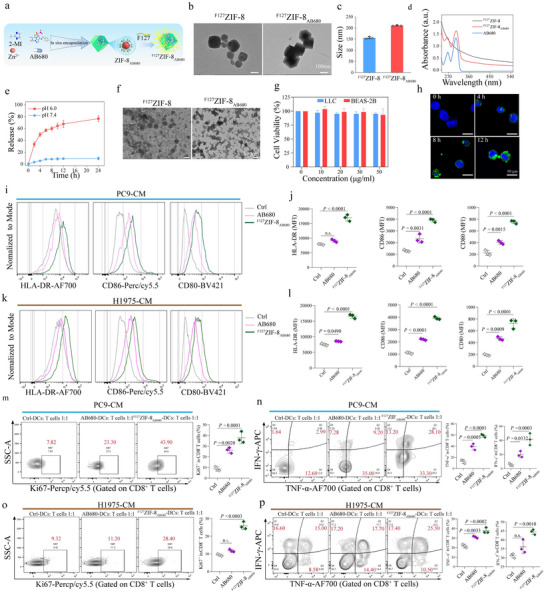
Schematic illustration of the fabrication and mechanism to impact DC function of ^F127^ZIF‐8_AB680_ nanoparticles (NPs) for cancer immunotherapy. a) Schematic illustration of the fabrication of ^F127^ZIF‐8_AB680_ NPs. b) Representative TEM image of ^F127^ZIF‐8 and ^F127^ZIF‐8_AB680_. c) The size of ^F127^ZIF‐8 and ^F127^ZIF‐8_AB680_. d) Absorbance of ^F127^ZIF‐8, AB680 and ^F127^ZIF‐8_AB680_. e) The accumulated Zn^2+^ ions release profile from ^F127^ZIF‐8 and ^F127^ZIF‐8_AB680_ NPs in PBS solutions with different pH values. f) TEM image of ^F127^ZIF‐8 and ^F127^ZIF‐8_AB680_ NPs treated with PBS solution (pH = 6.0) for 24 h. Scale bar, 100nm. g) Viability of the human bronchial epithelial cell line BEAS‐2B and mouse LLC after incubation with ^F127^ZIF‐8_AB680_ NPs at various concentrations for 24 h, respectively. h) Representative overlapping confocal laser scanning microscopy images of LLC incubated with ^F127^ZIF‐8_AB680_ NPs at various time points. Scale bar, 10µm. i–l) Immature DCs were cultured with CM from PC9 and H1975 cells treated with PBS, AB680, or ^F127^ZIF‐8_AB680_ for 48 h. Flow cytometry was used to assess the expression of maturation markers HLA‐DR, CD86, and CD80 on DCs, along with the quantification of mean fluorescence intensity. m–p) DCs cultured with CM from treated PC9 and H1975 cells were matured and co‐cultured with CD8^+^ T cells. Proliferation of CD8^+^ T cells (Ki67^+^) was evaluated, along with their ability to secrete TNF‐α and IFN‐γ.

In light of the critical role of CD73/adenosine in creating an immunosuppressive TME, previous preclinical and clinical efforts have been made in blockage of CD73/adenosine pathway using small‐molecule inhibitors in solid cancers, however, limited efficacy was reported mainly due to their low biostability and inefficient delivery in *vivo*.^[^ ^24^, ^25^ ^]^ To address these challenges, we engineered a pH‐responsive nanocarrier, ^F127^ZIF‐8_AB680_, designed to precisely deliver a selective CD73 inhibitor (AB680) to the acidic tumor areas. F127, a Pluronic block copolymer (PEO‐PPO‐PEO), was incorporated to enhance nanoparticle stability, improve aqueous dispersibility, and prevent aggregation, while ZIF‐8 provided a pH‐sensitive framework enabling controlled drug release in acidic environments. AB680, a potent small‐molecule inhibitor of CD73, served as the active therapeutic component. Together, these elements formed a nanocomposite system optimized for efficient and targeted delivery of AB680 (**Figure** **7**a). Under transmission electron microscopy (TEM) and scanning electron microscopy (SEM), ^F127^ZIF‐8_AB680_ nanoparticles (NPs) showed similar structures with the control NPs (^F127^ZIF‐8) (Figure 7b and Figure S11a, Supporting Information), whereas their size was slightly larger (200nm) than the control NPs (150nm) (Figure 7c). The absorption peaks of both ZIF‐8 and AB680 were simultaneously detected in the absorption spectrum of ^F127^ZIF‐8_AB680_, further confirming the successful loading of AB680 within ^F127^ZIF‐8 (Figure 7d). Moreover, there was no significant change in the zeta potential of the two materials, suggesting that the internal loading of AB680 does not affect the surface properties of the ^F127^ZIF‐8 (Figure S11b, Supporting Information). Further X‐ray diffraction (XRD) analysis revealed that the characteristic peaks of ^F127^ZIF‐8 were maintained after loading with AB680, indicating that the incorporation of the drug did not disrupt the crystalline structure of the ZIF‐8 framework, thereby preserving its structural stability and porosity (Figure S11c, Supporting Information). In addition, X‐ray photoelectron spectroscopy (XPS) analysis was also applied to test the compositions of samples. Among XPS high‐resolution scans of Zn 2p, O 1s, and N 1s peaks (Figure S11d,e, Supporting Information), no obvious shift was found in ^F127^ZIF‐8 and ^F127^ZIF‐8_AB680_ NPs, indicating a negligible effect of AB680 on the coordination of Zn^2+^ ions and 2‐methylimidazole.

As drugs carriers, ZIF‐8 NPs have been applied for loading molecules, enzymes, DNA and proteins, and are also capable of achieving pH‐responsive controlled release.^[^ ^33^ ^]^ To verify their pH‐responsive biodegradation and release, the constructed ^F127^ZIF‐8 or ^F127^ZIF‐8_AB680_ NPs were dispersed into PBS with different pH values (7.4 and 6.0). The accumulated Zn^2+^ ions release profile showed that the release rate of ^F127^ZIF‐8_AB680_ NPs under acidic condition (pH = 6.0) is up to 80% within 24 h, while under neutral condition (pH = 7.4), it is only about 10% (Figure 7e). Upon treatment with acidic PBS for 24 h, the ^F127^ZIF‐8 and ^F127^ZIF‐8_AB680_ NPs underwent obvious degradation, and no intact NPs can be detected under TEM and SEM (Figure 7f and Figure S11f, Supporting Information).

Next, we tested the biosafety of NPs, a prerequisite for their possibility in application. Different concentrations of ^F127^ZIF‐8_AB680_ were then incubated with mouse LLC tumor cells or human bronchial epithelial BEAS‐2B cells for 24 h, and clearly, ^F127^ZIF‐8_AB680_ was non‐toxic to both of them (Figure 7g). Further, as detected under confocal laser scanning microscopy (CLSM), the fluorescence of ^F127^ZIF‐8_AB680_ in LLC cells was apparently present after 4 h incubation, with signals continuing to rise up with the extension of incubation time, manifesting a robust uptake efficiency by tumor cells (Figure 7h).

Last, the in vivo biosecurity of ^F127^ZIF‐8_AB680_ NPs was systematically examined. Upon intravenous administration with ^F127^ZIF‐8_AB680_ NPs (10 mg kg^−1^) for 3 or 7 days, toxicological examinations including blood parameters, and hematoxylin and eosin (H&E) staining assays of the main organs were performed in mice, with no obvious inflammation and organ damages were detected (Figure S12a, Supporting Information). Further hemolysis assay showed no noticeable hemolytic activity in ^F127^ZIF‐8_AB680_ NPs‐treated groups even the NP concentration reaches up to 300 µg mL^−1^ (Figure S12b, Supporting Information). All these data strongly supported that our constructed ^F127^ZIF‐8_AB680_ possess good biocompatibility and biosecurity.

### 2.8 ^F127^ZIF‐8_AB680_ Reverses EGFR‐Mutant Tumor‐Induced DC Dysfunction In Vitro

With this newly constructed ^F127^ZIF‐8_AB680_, we next tested its potential modulation of CD73 expression and adenosine production in tumor cells, as well as its effects on anti‐tumor immunity. As expected, ^F127^ZIF‐8_AB680_ significantly suppressed CD73 expression on EGFR^mut^ human PC9 and H1975 cells (Figure S13a–d, Supporting Information), along with a corresponding reduction in adenosine production (Figure S13e, Supporting Information), validating that ^F127^ZIF‐8_AB680_ was functional in inhibition of the CD73/adenosine axis.

To determine the effect of ^F127^ZIF‐8_AB680_ on anti‐tumor immunity, the healthy human PB‐derived iDCs were cultured with CM from EGFR^mut^ human NSCLC cells (PC9 and H1975) followed with treatment by AB680 or ^F127^ZIF‐8_AB680_. Notably, ^F127^ZIF‐8_AB680_ was shown to be superior to AB680 in restoring the dysfunctional phenotype of DCs induced by EGFR^mut^ tumor cell products (Figure 7i–l). Consequently, DCs that were cultured with the ^F127^ZIF‐8_AB680_‐treated tumor CM displayed a marked increase in their ability to prime CD8^+^ T cell proliferation and activation, compared to the AB680‐treated groups or the CM control (Figure 7m–p). Therefore, ^F127^ZIF‐8_AB680_ showed a more potent capability than AB680 in the inhibition of CD73 expression and adenosine production in EGFR‐mutant NSCLC cells, leading to enhanced DC maturation and significantly improved CD8^+^ T cell activation.

### 2.9 ^F127^ZIF‐8_AB680_ Potently Improves the Efficacy of Anti‐PD‐1 in Treatment of in EGFR Mutant Tumor In Vivo

**Figure 8:**
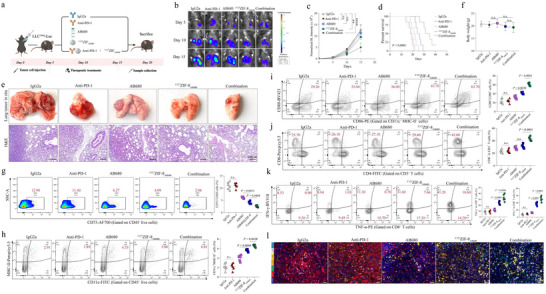
Antitumor Effects and Immune Modulation Induced by ^F127^ZIF‐8_AB680_ in an EGFR‐Mutant Lung Cancer Mouse Model. a) Schematic illustration of the treatment groups, including five distinct groups: IgG2a, Anti‐PD‐1, AB680, ^F127^ZIF‐8_AB680_, and ^F127^ZIF‐8_AB680_ combined with Anti‐PD‐1. (n = 6 samples per group) b) Representative bioluminescence imaging of tumor progression across the five groups. c) Quantitative analysis of tumor volume through fluorescence intensity measurements. d) Kaplan‐Meier survival curves comparing the different treatment groups. e) Representative images of lung tumors and corresponding H&E‐stained sections from each treatment group. Scale bar, 200µm. f) Mice weight measurements in different treatment groups. g) Flow cytometry analysis of CD73 expression in tumor tissues. h,i) Flow cytometry analysis of CD11c^+^MHC‐II^+^ DCs and mature DCs (CD86^+^CD80^+^) infiltrating tumor tissues. j,k) Flow cytometry analysis showing CD8^+^ T cells infiltration and their secretion of TNF‐α and IFN‐γ in tumor tissues. l) Representative images of mIF staining of tumor sections. Marker colors are as follows: CD73 (red), CD11c (green), MHC‐II (orange), CD86 (yellow), CD8 (white), and IFN‐γ (light blue). Scale bar, 50µm.

Inspired by the above promising in vitro results, we next determined whether ^F127^ZIF‐8_AB680_ could effectively remodel the immune TME and enhance the efficacy of immunotherapy in the syngeneic LLC tumor setting in *vivo*. To this end, the orthotopic LLC‐19del‐luciferase tumor‐bearing mice received 5 groups of treatment: vehicle (IgG2a), anti‐PD‐1, AB680, ^F127^ZIF‐8_AB680_, and ^F127^ZIF‐8_AB680_ + anti‐PD‐1 (combination) (**Figure** **8**a). Prior to these combination studies, we performed a dose‐ranging experiment to determine the optimal dose of ^F127^ZIF‐8_AB680_ in our model. Representative bioluminescent imaging and tumor growth curves are shown in Figure S14a,b, Supporting Information, confirming that 10 mg kg^−1^ was sufficient to achieve significant tumor suppression. Furthermore, pharmacokinetic and biodistribution analysis demonstrated that ^F127^ZIF‐8_AB680_ preferentially accumulated in tumor tissue compared to major organs, including heart, liver, spleen, lung, and kidney (Figure S14c,d, Supporting Information), supporting its effective delivery and tumor‐targeting capability.

While the conventional delivery of the CD73 inhibitor (AB680) did not show an effect, delivery of the drug with nanocarrier (^F127^ZIF‐8_AB680_) indeed significantly reduced the LLC tumor growth (Figure 8b,c). Importantly, in contrast to the insignificant effect of anti‐PD‐1 treatment alone, combination of anti‐PD‐1 with ^F127^ZIF‐8_AB680_ prominently delayed the growth of the orthotopic lung tumors and improved mouse survival (Figure 8b–e). Consistently, the combinational treatment led to remarkably reduced tumor cell proliferation in *vivo*, as shown by the Ki67 immunostaining of the tumor sections (Figure S16a, Supporting Information). Importantly, the NPs administration did not alter the body weights of the mice suggesting their biosecurity in vivo (Figure 8f). Thus, our in *vivo* results clearly indicated that delivery of the CD73 inhibitor with the pH‐responsive nanocarrier was superior to the conventional delivery method, and ^F127^ZIF‐8_AB680_ significantly enhanced the therapeutic efficacy of immune checkpoint blockade in treatment of EGFR mutant NSCLC.

### 2.10 ^F127^ZIF‐8_AB680_ Remodels the TME of NSCLC Tumors

To understand the mechanisms underlying the potent anti‐tumor effect of ^F127^ZIF‐8_AB680_, we first tested whether it was effective in an immunodeficient setting to preclude their possible modulation of other host systems than the adaptive immunity. In contrast to the immunocompetent setting, ^F127^ZIF‐8_AB680_ did not show a significant effect on restraining EGFR mutant LLC tumors in the mature T cell‐lacking nude mice in vivo (Figure S15, Supporting Information), suggesting that its anti‐tumor capacity is mainly dependent on regulation of the adaptive immunity.

Next, we characterized the immunomodulatory mechanisms of ^F127^ZIF‐8_AB680_. As expected, ^F127^ZIF‐8_AB680_ significantly repressed the adenosine production within the tumor tissues (Figure S16b, Supporting Information), as well as the ratio of CD73^+^ tumor cells (Figure 8g). Of note, the inhibitory effect of ^F127^ZIF‐8_AB680_ was more robust than that of AB680, suggesting a potentially selective delivery of the pH‐sensitive NPs to the acidic TME. By measuring the serum levels of immune regulatory cytokines including IL10, IL12p70, IFN‐γ and TNF‐α, it was shown that combination of ^F127^ZIF‐8_AB680_ with anti‐PD‐1 augmented the systemic production of anti‐tumor immunostimulatory cytokines (IL12p70, IFN‐γ and TNF‐α) while suppressing the immunosuppressive cytokine (IL‐10) indicating a systemic boosting of the anti‐tumor immune responses by the combinational therapy in EGFR‐mutant NSCLC in vivo (Figure S16c–f, Supporting Information).

Furthermore, considering the broad immunosuppressive role of adenosine, we assessed the effect of CD73 blockade on other key immunosuppressive populations within the TME. Flow cytometry analyses revealed that ^F127^ZIF‐8_AB680_ treatment tended to reduce the infiltration of Tregs (*p* = 0.0564) (Figure S17a, Supporting Information), monocytic MDSCs (M‐MDSCs; *p* = 0.0878), and polymorphonuclear MDSCs (PMN‐MDSCs; *p* = 0.0500) (Figure S17b, Supporting Information), while the infiltration of macrophages remained comparable between groups (Figure S17c, Supporting Information). These results suggest a potential, though modest, effect of CD73 inhibition on shaping multiple immunosuppressive cell subsets within the TME.

As shown earlier, DC dysfunction is characteristic of EGFR‐mutant NSCLC in both humans and mouse models. We therefore speculated that delivery of CD73 inhibitor by NPs may effectively reverse the DC functions within the TME. Indeed, ^F127^ZIF‐8_AB680_ showed a more potent effect than AB680 in increasing the infiltration of mature CD11c^+^MHC^+^DCs and CD80^+^CD86^+^DCs and consequently elevating the levels of TNF‐α^+^CD8^+^T and IFN‐γ^+^CD8^+^ effector T cells in the TME (Figure 8h–k). Compared to the anti‐PD‐1 and ^F127^ZIF‐8_AB680_ treatment alone, their combination demonstrated the highest degree of reversing the dysfunctional DCs and recruitment of the effector CD8^+^ T cells as shown by the flow cytometry and mIF staining (Figure 8h–l and Figure S18, Supporting Information), which was well consistent with the tumor response results. Collectively, in EGFR‐mutant preclinical models, delivery of the CD73 inhibitor with pH‐responsive NPs, superior to the conventional delivery approach, markedly remodels the immunosuppressive TME with augmented DC maturation and effector T cell infiltration, which is particularly effective when combining with immunotherapy.

## 3 Discussion

The clinical management of EGFR‐mutant NSCLC remains a significant challenge, as these patients exhibit markedly poor responses to ICIs compared to EGFR WT counterparts. Despite the transformative impact of ICIs on NSCLC treatment, EGFR mutations are consistently associated with diminished clinical benefit, and in some cases, even hyperprogressive disease.^[^ ^9^ ^]^ While previous studies have highlighted factors such as low TMB, reduced neoantigen presentation, and increased immunosuppressive cell populations like Tregs and MDSCs,^[^ ^13^, ^14^, ^15^ ^]^ these explanations fail to fully account for the profound immune resistance observed in EGFR‐mutant tumors. This underscores the need to investigate comprehensive mechanisms driving immune evasion in this patient population. By focusing on CD73‐adenosine axis and dysfunctional DCs, our study provides novel insights into how EGFR mutations reshape the TME to suppress the effective anti‐tumor immunity.

**Figure 9:**
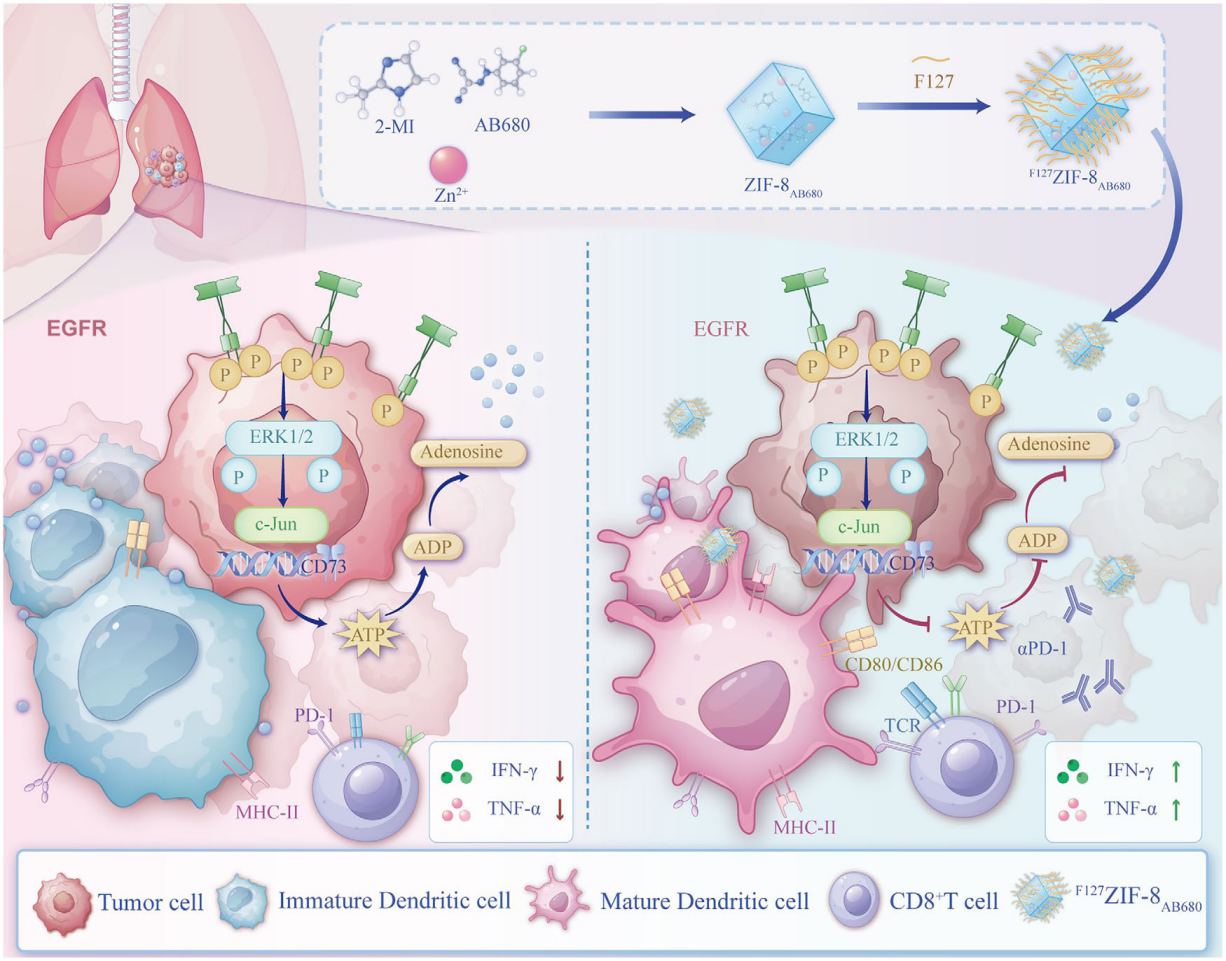
Mechanism of EGFR‐Driven Immune Suppression and ^F127^ZIF‐8_AB680_ Intervention in Tumor Immunity. This mechanistic diagram depicts the pathway by which EGFR mutations in tumor cells activate downstream ERK signaling, leading to c‐Jun expression and subsequent upregulation. c‐Jun binds to the CD73 promoter, initiating transcription and resulting in elevated adenosine levels in the tumor microenvironment (TME). High adenosine concentrations impair DC maturation, limiting antigen presentation to CD8^+^ T cells and thereby suppressing anti‐tumor immune responses. The pH‐responsive nanodrug – ^F127^ZIF‐8_AB680_ effectively targets CD73, reducing adenosine production in the acidic TME, enhancing DC maturation, and boosting CD8^+^ T cell activation, ultimately restoring anti‐tumor immunity.

In this study, we identified a novel mechanism of immune escape in EGFR‐mutant NSCLC, focusing on the critical role of DCs in anti‐tumor immunity. Our findings revealed that EGFR mutations disrupt DC maturation through the ERK/c‐Jun signaling axis, leading to CD73‐mediated adenosine accumulation in the TME. This adenosine‐rich environment suppresses DC function, impairing their ability to present antigens and prime CD8^+^ T cells, thereby weakening anti‐tumor immunity. By developing the pH‐responsive ^F127^ZIF‐8_AB680_, a nanodrug designed to precisely inhibit CD73 activity and reduce adenosine levels within the acidic TME, we demonstrated that it exerted a more potent effect than the conventional delivery method in restoration of DC maturation and functionality. This, in turn, enhanced CD8^+^ T cell activation and improved responses to PD‐1 blockade in preclinical models. These findings highlight DC dysfunction as a central element of EGFR‐driven immune evasion and underline the therapeutic potential of targeting DC maturation to overcome immune resistance in EGFR‐mutant NSCLC (**Figure** **9**).

Extensive evidence suggests that EGFR‐mutant cancer cells are a significant driver of immune suppression, actively shaping an immunosuppressive TME and impairing the quality of T cell‐mediated immune responses.^[^ ^31^, ^34^, ^35^, ^36^ ^]^ Moreover, tumor‐associated DCs usually exhibit immature phenotypes and functional impairment, which severely compromise their ability to initiate effective anti‐tumor immunity. These DCs often lack the necessary stimulatory signals or are actively rendered inactive by inhibitory mechanisms within the TME.^[^ ^37^, ^38^, ^39^ ^]^ However, few research has focused on the role of DCs in the EGFR mutation‐driven immunosuppressive microenvironment. Here, we evaluated the immune statuses of tumor tissues from EGFR‐mutant versus EGFR‐WT advanced NSCLC patients, as well as xenograft tumors through LLC lines transduced with the EGFR‐19del or EGFR‐WT gene and found that the EGFR mutations could fundamentally influence CD8^+^ T cells infiltration into tumor, which was consistent with previous studies.^[^ ^40^, ^41^ ^]^ What we found was that the functional loss of DCs might be a crucial factor for impeding CD8^+^ T cells infiltration into EGFR mutated tumors. Therefore, targeting EGFR mutations‐driven immune suppression to reverse DC function would be a promising strategy to enhance anti‐tumor immunity of CD8^+^ T cells and improve the efficacy of ICIs in this patient population.

Mechanistically, one of the most compelling aspects of this research is the demonstration of how EGFR mutations not only drive tumor progression through classical oncogenic signaling pathways but also profoundly reshape the TME by increasing adenosine production. In the last few decades, numerous studies have demonstrated that adenosine signaling involving CD39/CD73 ectonucleotidases expressed on tumor cells is a critical pathway in TME to evade the immune surveillance and generate an immunosuppressive milieu.^[^ ^42^, ^43^, ^44^ ^]^ Although the immunosuppressive role of adenosine has been established in various malignancies, including NSCLC, this study provides critical insights into how adenosine production in EGFR‐mutant TME disrupts DC functionality, representing a significant advance in understanding its impact on anti‐tumor immunity. Specifically, we demonstrate that EGFR mutations drive the overexpression of CD73, the enzyme responsible for adenosine production, via the ERK/c‐Jun signaling pathway. This pathway contributes to the creation of an immunosuppressive environment that impairs DC maturation and hinders their ability to activate CD8^+^ T cells. While prior studies have linked CD73 expression to EGFR‐driven signaling and poor prognosis in cancer patients,^[^ ^44^, ^45^, ^46^ ^]^ our findings delineate the precise molecular mechanism underlying CD73 upregulation in EGFR‐mutant NSCLC and confirming its pivotal role in shaping an immunosuppressive TME.

In comparison to previous studies, which have largely focused on inhibiting adenosine signaling through conventional delivery of small‐molecule inhibitors, this study goes a step further by incorporating a nanotechnology‐based delivery system to enhance drug stability and specificity. In the present study, we first make a critical advancement by synthesizing a novel nanodrug, ^F127^ZIF‐8_AB680_, which not only targets CD73 and reduces adenosine production but also exhibits enhanced stability, bioavailability, and tumor‐targeting properties. As drugs carriers, ZIF‐8 NPs have been applied to the loading of molecules, enzymes, DNA and proteins, achieving pH‐responsive controlled release in vivo.^[^ ^47^, ^48^, ^49^ ^]^ The pH‐sensitive nature of the ZIF‐8 framework allows for the controlled release of AB680, the CD73 inhibitor, directly within the acidic TME, thereby minimizing systemic toxicity and improving therapeutic efficacy. The application of ^F127^ZIF‐8_AB680_ in EGFR‐mutant NSCLC represents an innovative approach to overcome the limitations of current CD73 inhibitors, whose effects are compromised by poor tumor penetration and rapid clearance from the body. This nanodrug not only effectively overcomes the aforementioned limitations, but also significantly reverses the immunosuppressive effects of adenosine, allowing for improved DC maturation and subsequent activation of CD8^+^ T cells, which are critical for mounting an effective anti‐tumor immune response. More importantly, the combination of ^F127^ZIF‐8_AB680_ with PD‐1 blockade demonstrates a synergistic effect, restoring immune function and significantly reducing tumor burden in preclinical models of EGFR‐mutant NSCLC. Although the application of nanomedicine in cancer therapy has been explored,^[^ ^50^, ^51^, ^52^ ^]^ this study represents one of the first applications of a nanodrug targeting CD73 specifically in EGFR‐mutant lung cancer, providing a promising strategy to counteract the immunosuppressive TME and improve responses to ICIs in this hard‐to‐treat patient population.

Notably, recent advances in nanomedicine have further validated the therapeutic potential of targeting the adenosine pathway in combination with other modalities. Zhao et al.^[^ ^53^ ^]^ developed an AS1411 aptamer‐functionalized black phosphorus nanosystem co‐loaded with an A2AR inhibitor, which synergized photothermal therapy with adenosine blockade to enhance T cell infiltration and cytotoxicity in melanoma. Similarly, Li et al.^[^ ^54^ ^]^ constructed a biomimetic nanodrug encapsulating black phosphorus quantum dots and a CD73 inhibitor, which effectively suppressed adenosine production and augmented photothermal immunotherapy in lung cancer models. These studies, along with our present work, collectively underscore the promise of nanotechnology‐enabled adenosine pathway inhibition as a versatile strategy to reprogram the immunosuppressive TME and potentiate immunotherapy across different cancer types.

Despite the promising anti‐tumor effects of ^F127^ZIF‐8_AB680_ observed in preclinical models, several limitations should be acknowledged. Off‐target accumulation and systemic toxicity remain potential concerns, as nanoparticles can be taken up by non‐malignant tissues, and their long‐term safety in humans is not yet fully understood. Additionally, interspecies differences in pharmacokinetics and biodistribution may complicate direct extrapolation of dosing from mouse models to clinical settings. Addressing these challenges would be important for advancing the clinical translation of this strategy. Future studies could focus on optimizing tumor‐targeting properties, incorporating surface modifications to minimize non‐specific uptake, and performing comprehensive toxicological evaluations in larger animal models.

## 4 Conclusion

In summary, our study uncovers a crucial finding that DC maturation is markedly restricted within the TME of EGFR‐mutant NSCLC, which we identified as a significant barrier to effective anti‐tumor immunity. Mechanistically, we demonstrated that EGFR‐driven upregulation of CD73 promotes adenosine accumulation, an immunosuppressive factor that impairs DC maturation, thereby weakening their ability to prime CD8^+^ T cells and initiate robust anti‐tumor immune responses. Targeting this pathway with pH‐responsive ^F127^ZIF‐8_AB680_, nanoparticles to carry a CD73 inhibitor, effectively mitigated adenosine production within the acidic TME, thereby enhancing DC functionality and CD8^+^ T cell responses, highlighting a promising therapeutic approach for overcoming the immunotherapy resistance in EGFR‐mutant NSCLC.

## 5 Experimental Section

### Patients

Peripheral blood (PB) samples were collected from 20 patients with stage IIIB/IV NSCLC at Qilu Hospital of Shandong University between July 2023 and January 2024, including 11 patients with EGFR WT and 9 patients with EGFR mutant NSCLC patients. Peripheral blood mononuclear cells (PBMCs) were isolated from these blood samples using density gradient centrifugation. After lysing the red blood cells, PBMCs were cryopreserved in a vapor phase storage system in 90% fetal bovine serum (FBS) supplemented with 10% dimethyl sulfoxide (DMSO) until further analysis by flow cytometry. In addition, 52 tumor specimens were obtained from patients with advanced lung adenocarcinoma treated at Shandong Cancer Hospital between June 2012 and June 2020. All patients included in this study had not received therapy at the time of sample collection. Detailed patient characteristics are provided in Table S1, Supporting Information.

The study protocol was approved by the Institutional Ethics Committee of Shandong University Qilu Hospital (approval number: KYLL‐2018(KS)‐107) and adhered to the ethical principles outlined in the Declaration of Helsinki. Written informed consent was obtained from all participants prior to sample collection.

### Cell Culture

The human bronchial epithelial cell line BEAS‐2B, along with human lung cancer cell lines A549, SPCA1, H1299, H1650, PC9, H1975, HCC827, and the mouse LLC cell line (LLC), were obtained from the American Type Culture Collection (ATCC). All cell lines were cultured in either RPMI 1640 or Dulbecco's modified Eagle's medium (DMEM) (both from Gibco, Invitrogen), supplemented with 10% FBS (Gibco) and 1% penicillin‐streptomycin solution. Cells were maintained at 37 °C in a humidified incubator with 5% CO_2_. The EGFR mutation status of the human lung cancer cell lines was as follows: A549, SPCA1, and H1299 (EGFR WT); H1650, PC9, and HCC827 (EGFR exon 19 deletion); and H1975 (L858R and T790M double mutation).

### Lentivirus, Plasmid and siRNA Transfection

The EGFR overexpression plasmid and small interfering RNAs (siRNAs) targeting EGFR were obtained from GenePharma. Transfections of the EGFR overexpression plasmid and siRNAs were performed using Lipofectamine 2000 Transfection Reagent (Invitrogen; Cat No. 11 668 019) according to the manufacturer's protocol. Tumor cells were harvested 48 h post‐transfection for subsequent analyses. For the generation of stable transfectants, human EGFR (del746‐750aa) or EGFR WT lentiviral vectors were acquired from Shanghai GeneChem. Briefly, cells were seeded into 6‐well plates at a confluence of 70–80% and incubated overnight. A volume of 1 mL of fresh medium containing lentivirus was added to each well and incubated for 6 h, followed by replacement with fresh medium. After 72 h, successfully transduced cells were selected using 2 µg mL^−1^ puromycin (Sigma‐Aldrich; Cat No. P8833) to establish stable cell lines.

### Synthesis of ^F127^ZIF‐8_AB680_ NPs

The ^F127^ZIF‐8_AB680_ NPs were prepared using a two‐step method. Initially, ZIF‐8_AB680_ NPs encapsulating AB680 were prepared through the co‐assembly of Zn^2^⁺ and 2‐methylimidazole (2‐MI). Specifically, Zn (NO_3_)_2_·6H_2_O (10 mg) and AB680 (2 mg) were dissolved in 1 mL of deionized water, and stirred magnetically at 1200 rpm for 10 min. Subsequently, 100 mg of 2‐MI was dissolved in 1 mL of deionized water, which was then gradually added dropwise to the aforementioned solution. The mixture was stirred at room temperature for 15 min. The precipitate was collected by centrifugation and washed three times with methanol and deionized water, followed by drying under vacuum conditions.

In the second step, surface modification was performed to improve the biocompatibility of the ZIF‐8_AB680_ NPs. The prepared ZIF_AB680_ powder (10 mg) was dispersed into 5 mL deionized water, followed by the addition of 10 mg of Pluronic F127. The mixture was stirred overnight at room temperature. After centrifuging to collect the precipitate and washing it with deionized water, the product was finally vacuum‐dried to obtain ^F127^ZIF‐8_AB680_ NPs.

### Characterization of ^F127^ZIF‐8_AB680_ NPs

The morphology of ^F127^ZIF‐8 and ^F127^ZIF‐8_AB680_ were observed using transmission electron microscopy (TEM) and scanning electron microscopy (SEM), while their particle size and zeta potential were measured by Nanosizer. The UV–vis absorption spectrum of AB680, ^F127^ZIF‐8, and ^F127^ZIF‐8_AB680_ NPs were characterized by UV–vis spectrophotometer. X‐ray diffraction patterns and elemental composition of ^F127^ZIF‐8 and ^F127^ZIF‐8_AB680_ NPs were analyzed by in situ X‐ray diffractometer and X‐ray photoelectron spectroscopy, respectively.

### pH responsiveness of ^F127^ZIF‐8_AB680_ NPs

Disperse ^F127^ZIF‐8 and ^F127^ZIF‐8_AB680_ NPs uniformly into PBS solutions with pH values of 6.0 and 7.4, respectively, and incubate them at room temperature. At specified time intervals (0, 2, 4, 6, 8, 10, 12, and 24 h), centrifuge the solutions to separate and collect the supernatants. Utilize a UV–vis spectrophotometer to determine the concentration of AB680 present in the supernatants, and subsequently calculate its release rate over time. Furthermore, the morphologies of ^F127^ZIF‐8 and ^F127^ZIF‐8_AB680_ NPs, after being incubated in a pH 6.0 solution for 24 h, were examined using SEM.

### In Vitro Cytotoxicity

LLC and BEAS‐2B cells were seeded onto 96‐well plate and cultured for 12 h. Then, the cells were further incubated with ^F127^ZIF‐8_AB680_ NPs at different concentrations (0, 10, 20,30 and 50 µg mL^−1^) for another 24 h. After that, CCK‐8 reagent was added and incubated for 2 h. Finally, the absorbance value at 450 nm was measured and used to calculate the cell viability.

### Cell Uptake Experiment

To examine cellular uptake, confocal laser scanning microscopy (CLSM) was used to visualize the internalization of FITC‐labeled ^F127^ZIF‐8_AB680_ NPs in LLC cells. Cells were incubated with the NPs for various time points (0, 4, 8 and 12 h), followed by fixation, staining, and imaging.

### Hemolysis Analysis

Red blood cells freshly harvested from the blood of healthy mice underwent three rigorous washes with PBS solution (pH 7.4) before being incubated with FZ NPs at concentrations ranging from 30 to 300 µg mL^−1^ (specifically, 30, 60, 90, 120, 180, 240, and 300 µg mL^−1^) at a temperature of 37 °C for a period of 4 h. After this incubation, the solution was centrifuged at 2000 rpm for a duration of 10 min. Subsequently, the absorbance of the supernatant at a wavelength of 570 nm was meticulously measured to calculate the hemolysis rate.

### Animal Study

All animal experiments were conducted in compliance with guidelines approved by the Institutional Animal Care and Use Committee of Shandong University Qilu Hospital (permit number: DWLL‐2024‐036). 6‐to‐8‐week‐old female C57BL/6J mice and female nude mice were obtained from Beijing Vital River Laboratory Animal Technology Company. All mice were housed in a pathogen‐free facility, maintained on a 12‐h light/12‐h dark cycle, with free access to food and water.

For the C57BL/6J mice model, luciferase‐expressing WT LLC (WT/LLC) and EGFR exon 19 deletion‐mutant LLC (19del/LLC) cells were used to establish lung tumors. A total of 5 × 10⁵ cells from each cell line were inoculated via trans‐thoracic injection into the lungs of the mice on day 0. Anti‐mouse PD‐1 antibody (200 µg per mouse) or control IgG2a (200 µg per mouse) was administered intraperitoneally (i.p.) every 3 days starting from day 5 post‐tumor inoculation, for a total of four injections. Simultaneously, mice were divided into three treatment groups: saline control, AB680 (10 mg kg^−1^), and ^F127^ZIF‐8_AB680_ NPs (10 mg kg^−1^). These treatments were administered intravenously (i.v.) via tail vein on the same schedule (four doses every 3 days starting from day 5 post‐inoculation). Tumor growth was assessed on days 5, 10, and 15 using bioluminescence imaging (IVIS spectrum in vivo imaging system, PerkinElmer, Hopkinton, MA, USA). On day 20, all mice were euthanized, and lung tumors were excised and weighed for further analysis. In addition, a survival study was conducted using the same treatment regimen. Mice were monitored for survival, and survival curves were plotted to assess the effects of the treatments. The in vivo distribution and tumor‐targeting capability of ^F127^ZIF‐8_AB680_ were evaluated using an orthotopic tumor model. Rhodamine‐labeled ^F127^ZIF‐8_AB680_was intravenously injected into tumor‐bearing mice at a dose of 10 mg kg^−1^. The biodistribution was monitored non‐invasively at 0, 12, 24, 48, and 72 h post‐injection using an in vivo imaging system. Following the final imaging time point, major organs (heart, liver, spleen, lung, kidney) and tumor tissues were harvested for *ex vivo* fluorescence quantification to quantitatively assess nanoparticle accumulation.

To further investigate whether the inhibitory effect of ^F127^ZIF‐8_AB680_ NPs was immune‐independent, a nude mouse model was employed. Nude mice (6–8 weeks old) were subcutaneously inoculated with 5 × 10⁵ 19del/LLC cells in the dorsal flank. Once the tumors reached a measurable size, the mice were randomly assigned to one of three groups: saline control, AB680 (10 mg kg^−1^), or ^F127^ZIF‐8_AB680_ NPs (10 mg kg^−1^). Treatments were administered via tail vein injection every 3 days, for a total of four doses, following the same schedule as in the C57BL/6J model. Tumor size was measured every 3 days using calipers, and tumor volume was calculated using the formula: volume = (length × width^2^) / 2. At the experimental endpoint, tumors were harvested and weighed for further analysis.

### In Vitro Generation of Human DCs

PBMCs were isolated from healthy volunteers using Ficoll‐Paque density gradient centrifugation, followed by magnetic separation of CD14⁺ monocytes using CD14 MicroBeads (Miltenyi Biotec, Cat No. 130‐050‐201). To generate DCs, CD14⁺ monocytes were cultured in RPMI 1640 medium supplemented with 10% FBS, GM‐CSF (1000 U mL^−1^), and IL‐4 (500 U mL^−1^) (R&D Systems). The culture medium was refreshed every 48 h. For direct exposure experiments, CD14⁺ cells were treated with conditioned media (CM) from EGFR mutant or WT NSCLC cell lines from day 2 to evaluate their impact on DC differentiation over 5 days. For maturation assays, monocytes were first cultured in GM‐CSF and IL‐4 for 5 days, followed by stimulation with lipopolysaccharide (LPS, 10 ng mL^−1^, Sigma‐Aldrich) in combination with CM from EGFR mutant or WT cells to assess the effect on DC maturation. Additionally, CM from EGFR mutant NSCLC cells (PC9 and H1975) pre‐treated with AB680 or ^F127^ZIF‐8_AB680_ NPs were used to culture immature DCs to evaluate potential improvements in DC maturation.

### DCs‐Driven CD8⁺ T Cell Activation in Mixed Lymphocyte Reaction

CD8⁺ T cells were isolated from healthy human PB using a negative selection kit (Miltenyi Biotec) according to the manufacturer's protocol. DCs, generated from CD14⁺ monocytes as described, were treated with CM from EGFR‐mutant or WT NSCLC cells, as well as CM from cells pre‐treated with AB680 or ^F127^ZIF‐8_AB680_. The treated DCs were then co‐cultured with CD8⁺ T cells at a 1:1 ratio (DCs:CD8⁺ T cells) for 48 h. CD8⁺ T cell proliferation was assessed by flow cytometry through the detection of Ki67 expression. Additionally, the functional activity of CD8⁺ T cells was evaluated by measuring the secretion of IFN‐γ and TNF‐α using intracellular cytokine staining and flow cytometry.

### Flow Cytometry and Abs

Excised tumors were minced and digested in RPMI 1640 medium containing collagenase IV (Worthington) and deoxyribonuclease I (DNase, Sigma‐Aldrich) at 37 °C for 40 min on a rotator. The digested tumor tissue was then passed through a 70µm strainer to obtain single‐cell suspensions, followed by red blood cell lysis. To block nonspecific binding, cells were pre‐incubated with anti‐CD16/CD32 (BioLegend) at 4 °C for 15 min before staining.

Surface marker staining was performed on both tumor‐derived cells and in vitro cultured cells. DCs were stained for HLA‐DR, CD86, and CD80 to assess their maturation status, while CD8⁺ T cell activation was evaluated by Ki67 expression for proliferation and intracellular staining of TNF‐α and IFN‐γ for functional assays. Additionally, CD73 expression on tumor cells was analyzed by flow cytometry to assess the effect of treatments on the adenosine pathway. All surface and intracellular staining was conducted using specific fluorophore‐conjugated antibodies (Table S2, Supporting Information). Viability of cells was assessed using Zombie Aqua Fixable Viability Dye (BioLegend, 1:400 dilution) prior to staining. Flow cytometry was conducted using a BD FACS Celesta system, and data were analyzed using FlowJo software.

### Enzyme‐Linked Immunosorbent Assay

To measure serum levels of IL‐12P70, IL‐10, TNF‐α, and IFN‐γ, blood samples were collected from mice via retro‐orbital bleeding and allowed to clot for 30 min at room temperature. Serum was separated by centrifugation at 1500 g for 10 min and stored at ‐80 °C until analysis. Enzyme‐linked immunosorbent assay (ELISA) kits specific for each cytokine were used according to the manufacturer's protocols (Multi Sciences). Absorbance was measured at 450 nm using a microplate reader, and cytokine concentrations were calculated based on standard curves generated with known concentrations of each cytokine.

### ATP and Adenosine Measurement

ATP and Adenosine levels were measured in cell culture supernatants and tumor tissue homogenates. Supernatants were collected from EGFR‐mutant and WT cell lines, including those treated with AB680 or ^F127^ZIF‐8_AB680_ NPs, and immediately processed. Tumor tissues were excised, homogenized in ice‐cold PBS, and directly analyzed. Both supernatants and homogenates were processed using a commercial ATP Assay Kit (Beyotime) and Adenosine Assay Kit (Biovision) following the manufacturer's protocol. ATP and Adenosine levels were quantified via fluorescence detection, and data were normalized to protein concentration using the Bradford assay.

### Quantitative RT‐qPCR Analysis

Tumor cells were harvested, and total RNA was extracted using Trizol (Invitrogen) according to the manufacturer's instructions. cDNA was synthesized from 2 µg of purified total RNA using the Evo M‐MLV RT Mix Kit (Accurate Biotechnology). The mRNA expression levels of CD73, EGFR, and c‐Jun were determined using specific primers and quantified by the ΔΔCt method, normalized to β‐actin as an internal control. All experiments were performed in triplicate, and the specific primers are listed in Table S3, Supporting Information.

### Immunoblotting Analysis

RIPA lysis buffer (Beyotime) with 1 mM phenylmethanesulfonyl fluoride (Beyotime) was used to lyse cells following centrifugation at 12 000 × g for 15 min. Protein concentrations were measured using Enhanced BCA Protein Assay Kit (Beyotime). Cell lysates were prepared from lung cancer and separated by 8–12% SDS‐PAGE. Then, the separated proteins were transferred onto PVDF membrane (Millipore). Membranes were blocked with 5% milk or BSA in TBST for 2 h at room temperature and incubated primary Abs overnight at 4 °C. The membranes were washed three times with TBST, incubated with HRP‐conjugated secondary Abs for 1 h at room temperature. After three times washes with TBST, the membranes were visualized by ECL (Millipore). The used primary and secondary antibodies information were list in Table S4, Supporting Information.

### CUT&RUN‐qPCR Assay

To explore the binding interactions between c‐Jun and the CD73 promoter, a CUT&RUN‐qPCR assay was performed using the Vazyme HD101 kit, following the manufacturer's instructions. A549 WT/19del cells were permeabilized and incubated with primary antibodies against the transcription factor of interest. Concanavalin A beads immobilized the nuclei, followed by cleavage of the chromatin using protein A‐MNase at sites bound by the transcription factor. The released DNA fragments were purified and analyzed by quantitative PCR (qPCR) to assess the enrichment at specific promoter regions, with the following CD73 promoter primers:

Primer 1^#^: Forward primer: CAGGCAATGAGAAAGGCACAT
Reverse primer: TCCTGTGTTCTTAGCCTCCT
Primer 2^#^: Forward primer: GGGAACAACCTCTCCACTCTT
Reverse primer: GCGAGTAGGGGCGAGC

Additionally, gel electrophoresis was used to confirm the specificity of the primers before qPCR analysis, ensuring accurate detection of promoter binding events.

### Dual Luciferase Reporter Assay

A PGL3 vector containing the CD73 promoter region upstream of the luciferase gene was generated. Luciferase activity was analyzed using Luciferase Assay System (Promega). HEK‐293T cells plated in 12‐well plates were co‐transfected 1 µg of PGL3‐CD73 promoter and 1µg of overexpressed c‐Jun construct and 0.1µg of control Renillla construct using Lipofection 2000 transfection reagent (Invitrogen). After 24 h of transfection, the luciferase activity was measured using Luciferase Assay system (Promega) and a microplate reader according to the manufacturer's instructions. Firefly luciferase activity was normalized to Renilla luciferase activity.

### Immunohistochemistry and Multiplex Immunofluorescence

For IHC, tumor tissues were fixed in 10% formalin, embedded in paraffin, and sectioned at 4 µm thickness. Sections were deparaffinized, rehydrated, and subjected to antigen retrieval using citrate buffer (pH 6.0) in a microwave. Endogenous peroxidase activity was blocked with 3% hydrogen peroxide, and non‐specific binding was blocked with 5% bovine serum albumin (BSA). Sections were incubated with primary antibodies overnight at 4 °C, followed by incubation with biotinylated secondary antibodies and streptavidin‐HRP. Visualization was achieved using DAB substrate, and sections were counterstained with hematoxylin.

For mIF staining, tumor sections were similarly prepared and antigen retrieval was performed. Sections were blocked with 5% BSA and incubated sequentially with primary antibodies specific for different markers, each followed by corresponding fluorescently labeled secondary antibodies. Nuclei were stained with DAPI. Slides were mounted with antifade medium and imaged using a fluorescence microscope. Quantification was performed using image analysis software to measure the intensity and localization of each marker. The specific antibodies used for IHC and mIF are listed in Table S5, Supporting Information.

### Liquid Chromatography‐Mass Spectrometry Analysis

Metabolites were extracted from 15 mL of supernatants collected from A549/19del and A549/WT cells, following the manufacturer's protocol for LC‐MS analysis. The metabolomic profiling was performed in collaboration with Shanghai Bioprofile Technology Co., Ltd (Shanghai, China). To ensure data quality and consistency during metabolic profiling, quality control (QC) samples were prepared by pooling aliquots from all samples under analysis. These QC samples were used for data normalization. Metabolites were detected using electrospray ionization in both negative and positive modes. The raw multiple reaction monitoring (MRM) data for each metabolite were extracted and quantified using MultiQuant 3.0.2 software, generating the peak areas for each metabolite. Differential metabolites were subjected to KEGG pathway enrichment analysis using the KEGG database (http://kegg.jp). The identified differential metabolites are listed in Table S6, Supporting Information.

### Statistical Methods

Group comparisons were performed using one‐way or two‐way ANOVA with post hoc tests. Kaplan‐Meier survival analysis was evaluated using the log‐rank test, and correlation analyses were conducted using Pearson's correlation. Forest plots were generated to visualize effect sizes and confidence intervals. The scRNA‐seq data from GSE131907 and TCGA datasets were analyzed to compare immune cell infiltration and CD73 gene expression differences between EGFR‐mutant and WT patients. Statistical significance was set at *p* < 0.05, with analyses performed using GraphPad Prism 8.0 or R packages 4.1.

## Conflict of Interest

The authors declare no conflict of interest.

## Author Contributions

Y.L. and X.W. conceived the project and designed the study. X.S. performed the in *vivo* and in vitro studies, generated the figures, statistical analysis and wrote the manuscript. X.G. performed in vitro studies of NPs. Z.W. and S.Y. assisted in the construction of mouse lung tumor model in situ. S.D. collected patients’ paraffin tissue samples and conducted clinical follow‐up with relevant information. N.L. and X.M. helped with the in vitro experiments. X.S., H.W., and Y.S. helped with the in *vivo* experiments. X.Q. guided the experimental design. G.R. performed critical assistance on experimental design and manuscript writing. Y.L. guided the NPs design and manuscript revision in this project.

## Supporting information



Supporting Information

## Data Availability

The data that support the findings of this study are available from the corresponding author upon reasonable request.
