# Supplementary material for: Overcoming EGFR‐Mediated Dendritic Cell Dysfunction to Enhance Anti‐tumor Immunity in EGFR‐Mutant NSCLC by Precisely Targeting CD73 With pH‐responsive Nanocarriers
Source: Adv Sci (Weinh). 2025 Oct 27;13(1):e13182. doi: 10.1002/advs.202513182 (PMC12767102; doi:10.1002/advs.202513182)
Supplement: Supplementary file 1 — Supporting Information [file ADVS-13-e13182-s001.docx]

**Title:** Overcoming EGFR-Mediated Dendritic Cell Dysfunction to Enhance Anti-tumor Immunity in EGFR-mutant NSCLC by Precisely Targeting CD73 with pH-responsive Nanocarriers

**Author order:** Xiaoling Shang^1^, Xudong Geng^3^, Zixu Wang^1^, Shumin Yuan^1^, Shanshan Ding^4^, Ni Liu^1^, Xinchun Ma^1^, Xuan Sun^1^, Huimin Wang^1,5^, Ying Sun^1^, Xun Qu^6^, Guangwen Ren^2^, Yong-Qiang Li^3^, Xiuwen Wang^1,7^* and Yanguo Liu^1,7^*

**Affiliations:**

^1^Department of Medical Oncology, Qilu Hospital of Shandong University, 107 Wenhuaxi Road, Jinan, Shandong, 250012, China.

^2^The Jackson Laboratory, Bar Harbor, Maine 04609, USA.

^3^Institute of Advanced Interdisciplinary Science, School of Physics, Shandong University, Jinan, Shandong, 250100, China.

^4^Department of Clinical Laboratory, Shandong Cancer Hospital & Institute, Shandong First Medical University & Shandong Academy of Medical Sciences, Jinan, Shandong, 250017, China.

^5^Department of Oncology, Yantai Affiliated Hospital of Binzhou Medical University, Yantai, Shandong, 264100, China.

^6^Laboratory of Basic Medical Sciences, Qilu Hospital of Shandong University, 107 Wenhuaxi Road, Jinan, Shandong, 250012, China.

^7^Lung Cancer Center, Qilu Hospital of Shandong University, 107 Wenhuaxi Road, Jinan, Shandong, 250012, China.

***Correspondence:**

Xiuwen Wang

Electronic address: [xiuwenwang12@sdu.edu.cn](mailto:xiuwenwang12@sdu.edu.cn)

Yanguo Liu

Electronic address: [liuyanguo@sdu.edu.cn](mailto:liuyanguo@sdu.edu.cn)

Tel: 860531-82169851; Fax: 860531-82169851


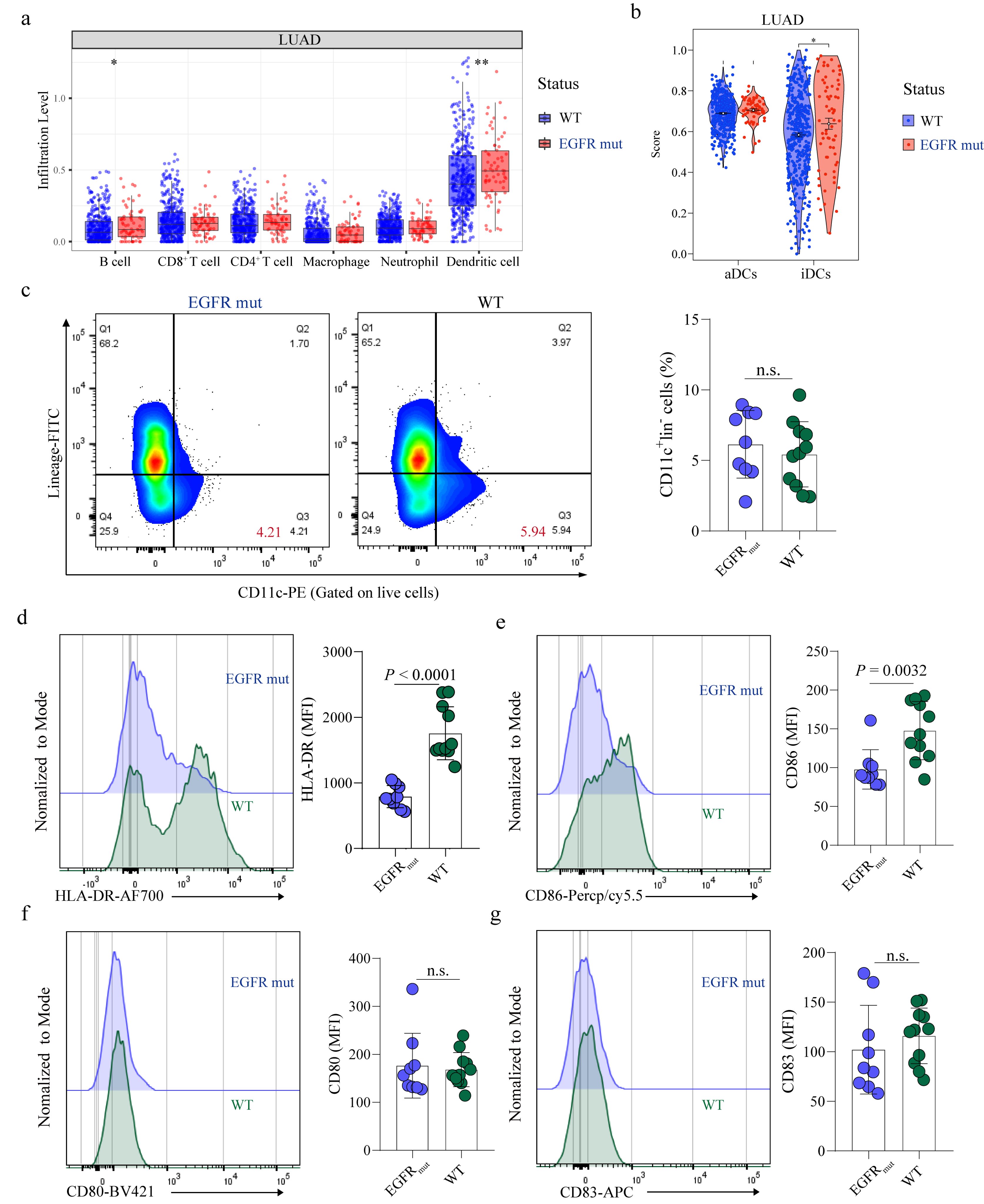


**Figure S1. Immune cell infiltration and DC phenotype analysis in EGFR-mutant and WT NSCLC patients. a** Analysis of immune cell infiltration in EGFR-mutant and WT NSCLC tumors using TIMER. **b** Proportion of different DC subtypes, including activated DCs (aDCs), immature DCs (iDCs), and plasmacytoid DCs (pDCs), in the TME of EGFR-mutant and WT NSCLC patients. **c** Flow cytometry analysis of the percentage of CD11c^+^lin^-^ cells in the peripheral blood of 9 EGFR-mutant and 11 WT NSCLC patients. **d-e** Flow cytometry analysis of HLA-DR and CD86 expression on DCs from the peripheral blood of 9 EGFR-mutant and 11 WT patients. **f-g** Flow cytometry analysis of CD80 and CD83 expression on peripheral blood DCs from the same patient cohort. **P* < 0.05, n.s., no significance.


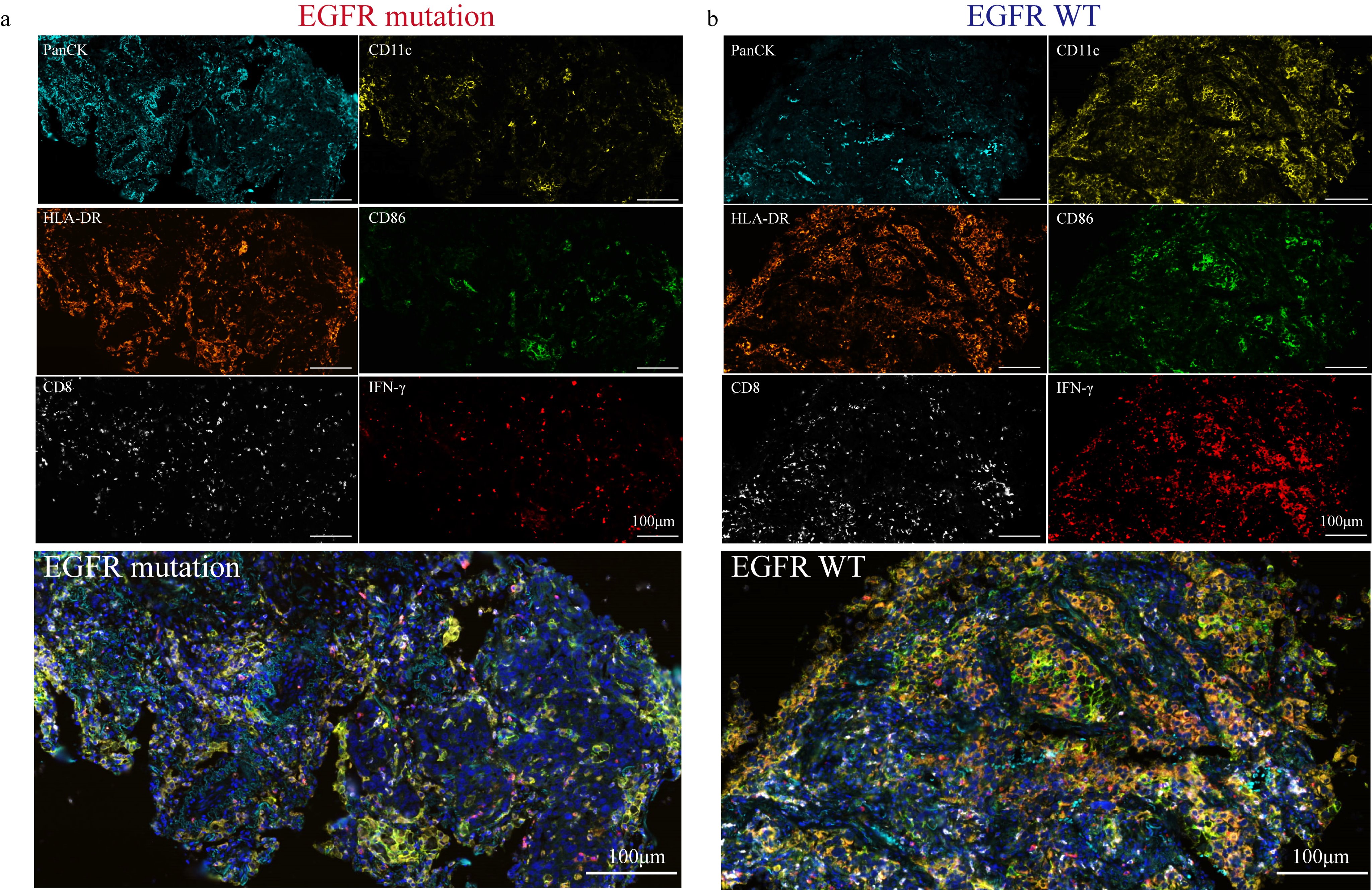


**Figure S2. The single-channel immunofluorescence staining used in the multiplex staining.** Fluorescence signals for PanCK (in light blue), CD11c (in yellow), HLA-DR (in orange), CD86 (in green), CD8 (in white), and IFN-γ (in red) are presented in EGFR mutation (a) and EGFR WT (b) patients. Scale bar, 100μm.


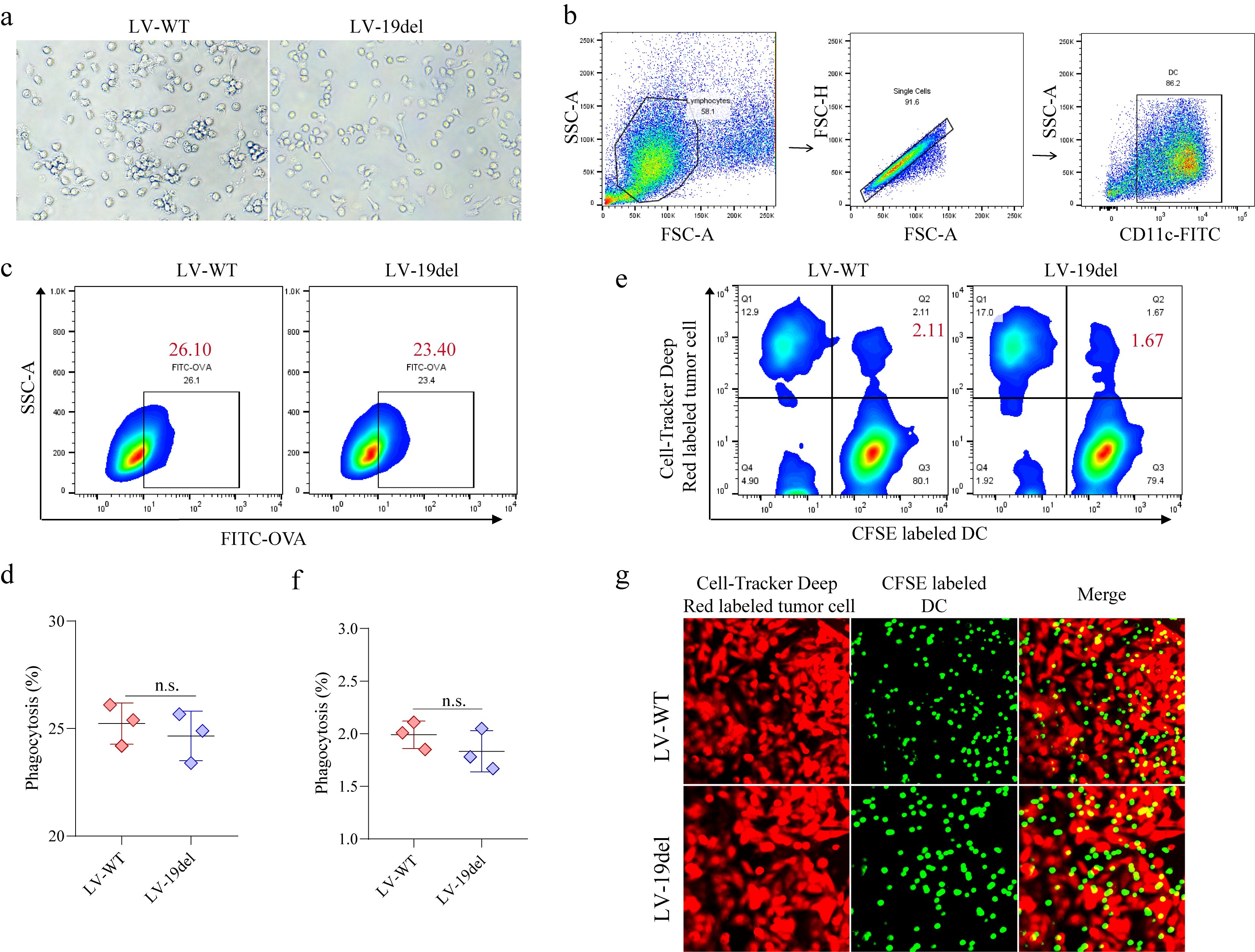


**Figure S3. Assessment of DC maturation and phagocytic function in response to EGFR-mutant and WT CM. a** Microscopic analysis of DCs after differentiation from iDCs to mature DCs, comparing the impact of EGFR-mutant and WT CM on their maturation capacity. **b** Flow cytometry gating strategy used to assess the phenotype of DCs. **c-d** Quantification of FITC-OVA uptake by iDCs cultured with CM from EGFR-mutant and WT cells, indicating the phagocytic activity of iDCs. **e-f** Flow cytometry analysis of the ability of iDCs cultured in EGFR-mutant and WT CM to phagocytose tumor cells. **g** Confocal microscopy demonstrating the interaction between iDCs and tumor cells, with tumor cells labeled in red and iDCs in green. n.s., no significance.


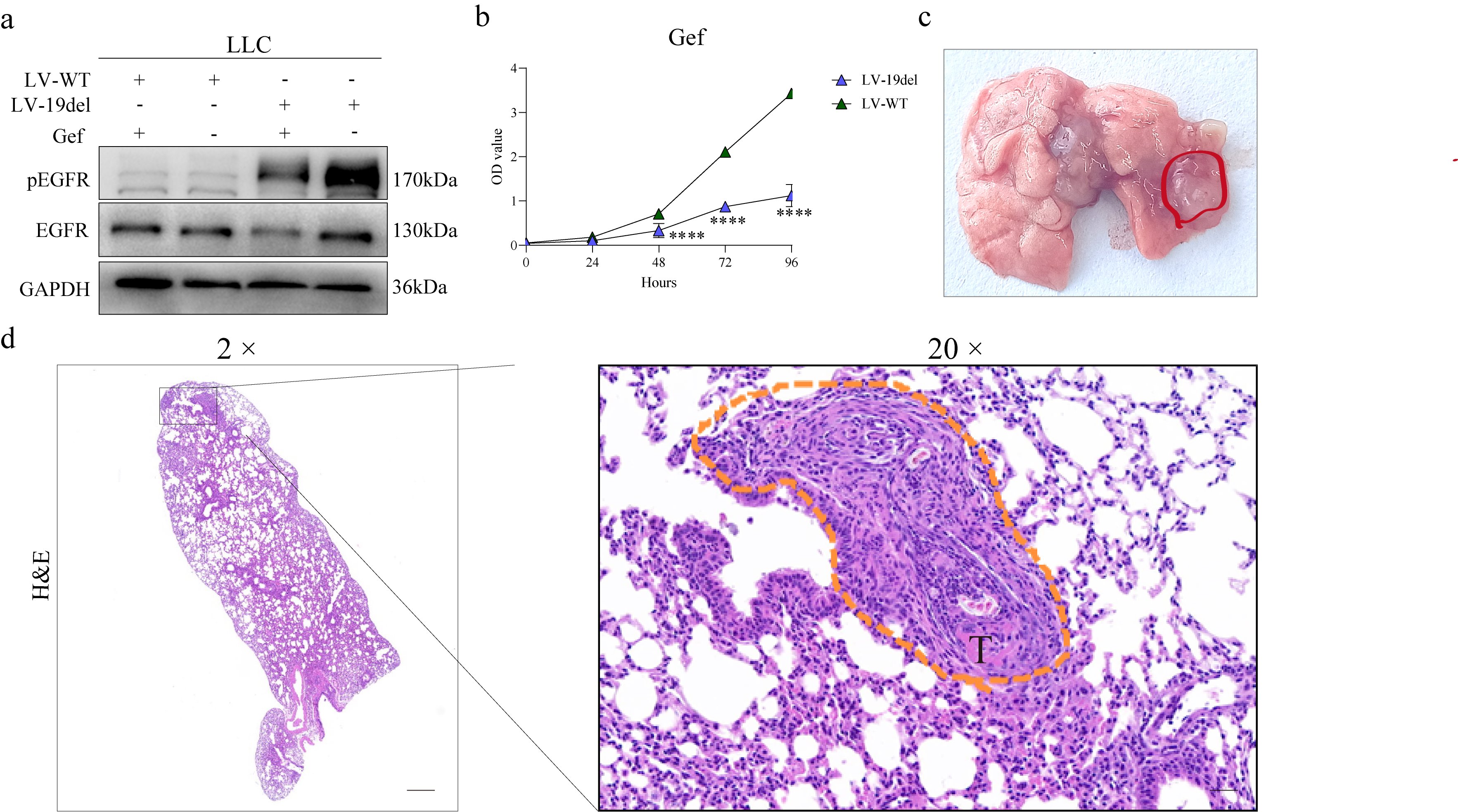


**Figure S4. Construction of EGFR-mutant LLC cell model and orthotopic lung tumor Model in C57BL/6J mice. a** Immunoblot analysis of EGFR and pEGFR in LLC cells transfected with human EGFR-19del or WT, treated with or without gefitinib. **b** CCK-8 assay showing gefitinib sensitivity of LLC cells expressing EGFR-19del or WT. **c** Representative images of orthotopic lung tumors in C57BL/6J mice transplanted with LLC cells expressing EGFR-19del or WT. **d** H&E staining of the lung tumors, with "T" indicating tumor regions. *****P* < 0.0001.


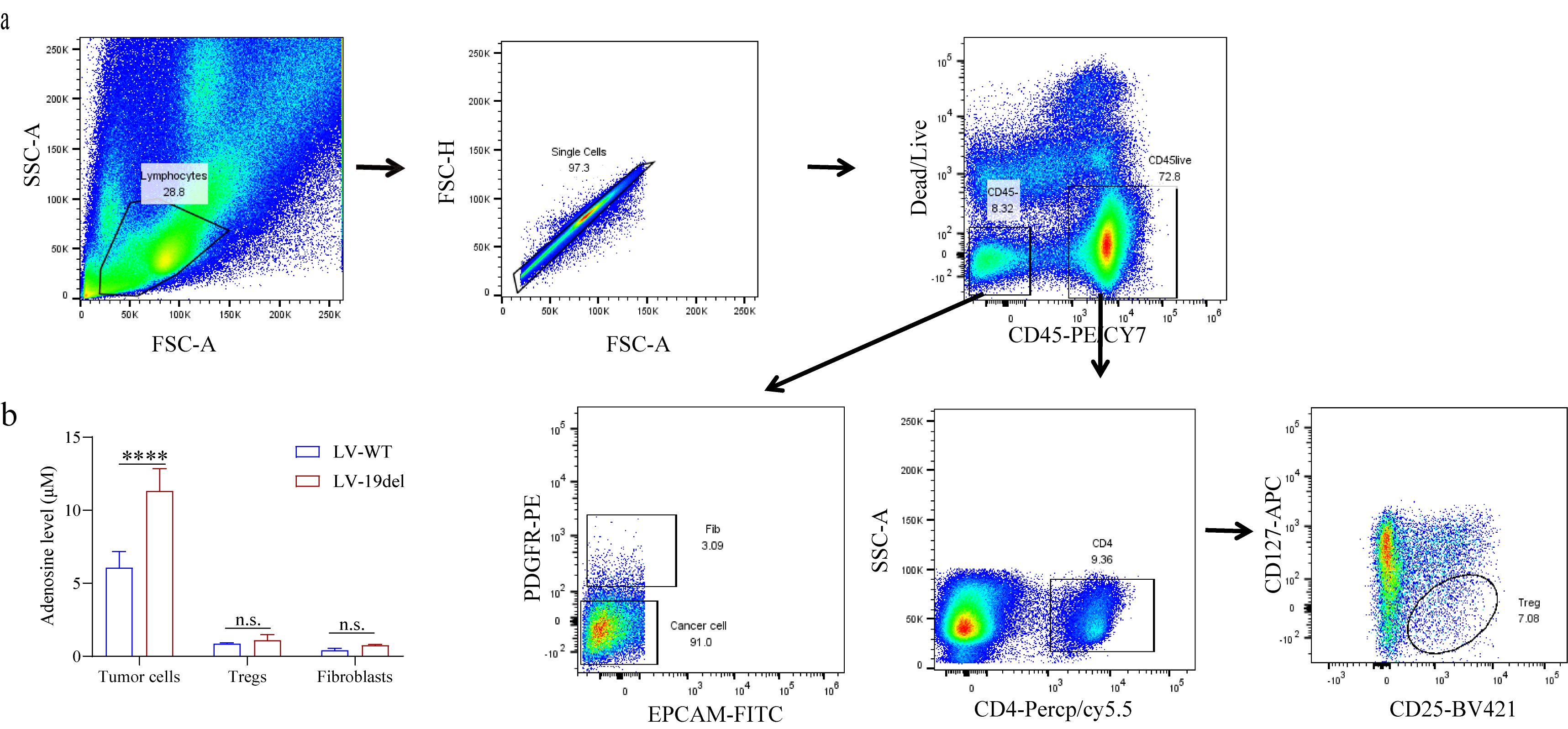


**Figure S5. Identification of cellular sources of adenosine in orthotopic lung tumors.** **a** Flow cytometry gating strategy for sorting tumor cells, fibroblasts (PDGFR^+^CD45^-^cells), and regulatory T cells (CD25^high^CD127^low^ in CD4^+^T cells) from orthotopic lung tumor tissues. **b** Adenosine levels in each sorted population measured using a commercial adenosine detection kit. *****P* < 0.0001, n.s., no significance.

**
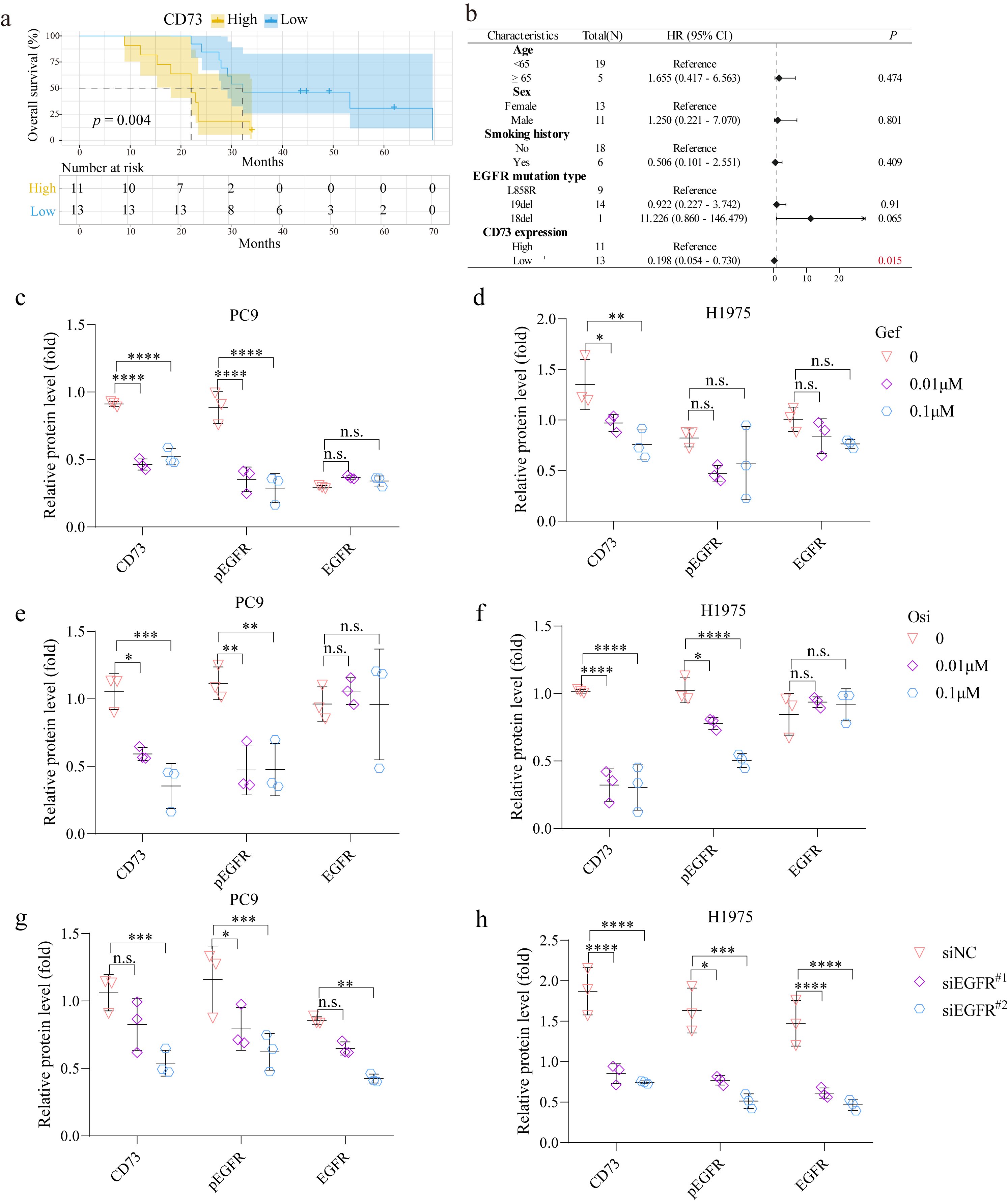
**

Figure S6. CD73 expression is regulated by EGFR in NSCLC and correlates with poor prognosis. **a** Kaplan-Meier survival curves comparing overall survival between low and high CD73 expression groups in 24 EGFR-mutant NSCLC patients. **b** Forest plot indicating that CD73 is an independent prognostic factor for poor survival in EGFR-mutant NSCLC patients. **c-f** Quantification of CD73, EGFR, and phosphorylated EGFR protein levels in PC9 and H1975 cells treated with gefitinib, osimertinib determined by densitometric analysis of immunoblotting results. **g-h** Quantification of CD73, EGFR, and phosphorylated EGFR protein levels following EGFR knockdown in PC9 and H1975 cells. **P* < 0.05, ***P* < 0.01, ****P* < 0.001, *****P* < 0.0001, n.s., no significance.

**
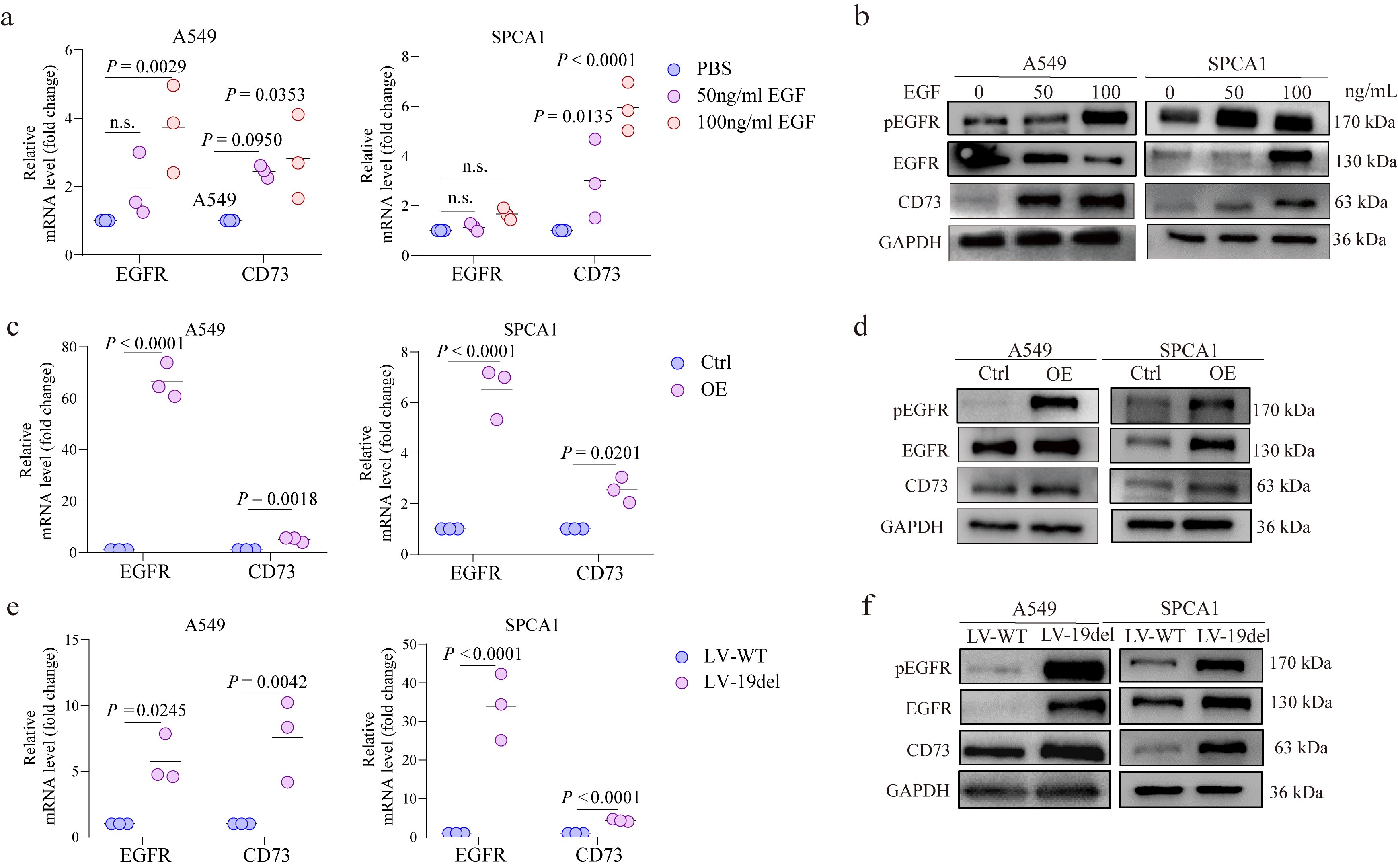
**

**Figure S7. EGFR activation regulates CD73 expression in A549 and SPCA1 cells. a** RT-qPCR analysis of EGFR and CD73 mRNA levels in A549 and SPCA1 cells treated with varying concentrations of EGF. **b** Immunoblot analysis of CD73, EGFR, and phosphorylated EGFR (pEGFR) in A549 and SPCA1 cells following EGF treatment. **c** RT-qPCR analysis of EGFR and CD73 mRNA expression in A549 and SPCA1 cells transfected with EGFR overexpression plasmids. **d** Immunoblot analysis of CD73, EGFR, and pEGFR in A549 and SPCA1 cells transfected with EGFR overexpression plasmids. **e** RT-qPCR analysis of EGFR and CD73 mRNA in A549 and SPCA1 cells transduced with EGFR 19del virus. **f** Immunoblot analysis of CD73, EGFR, and pEGFR in A549 and SPCA1 cells transduced with EGFR 19del virus.


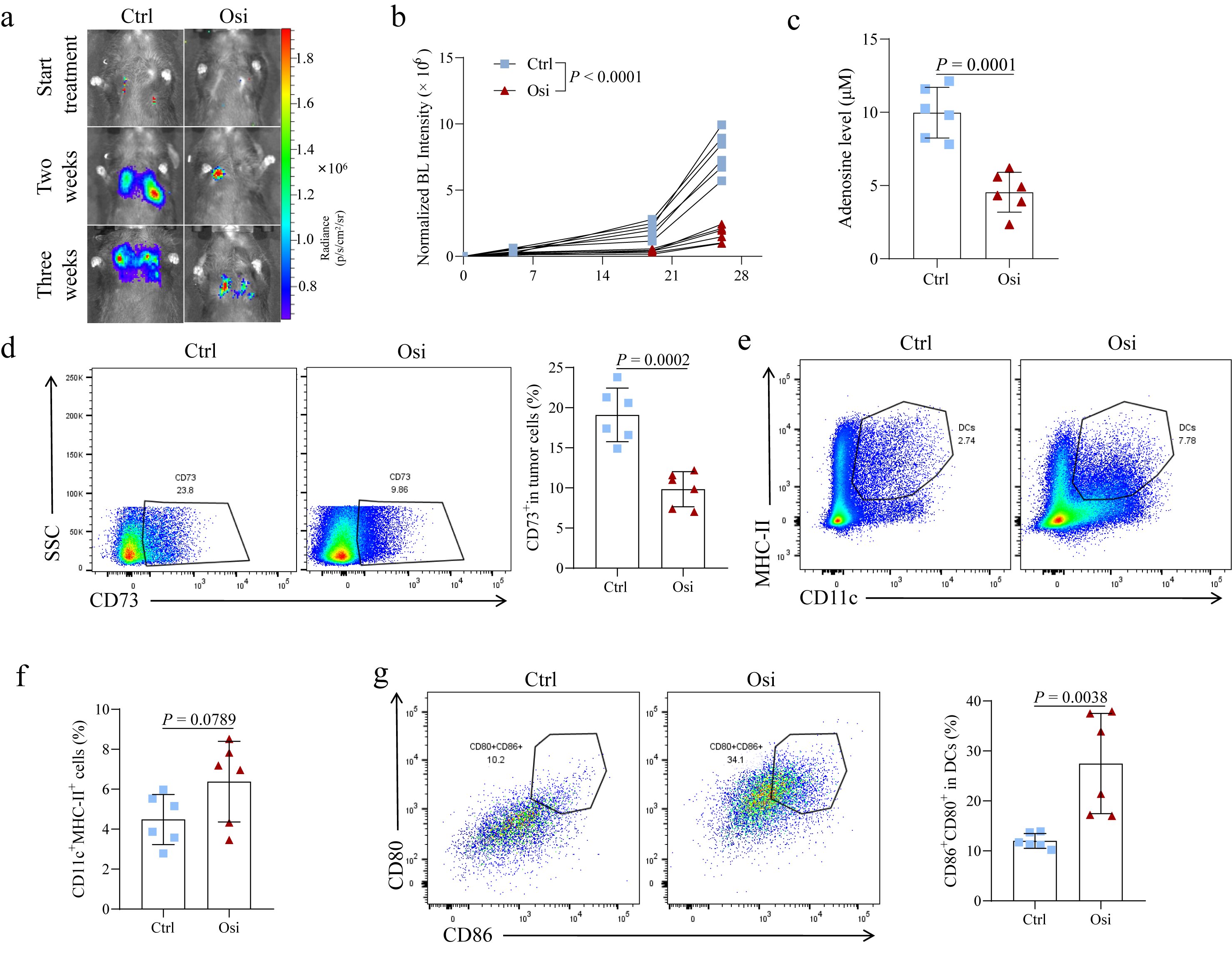


**Figure S8. Osimertinib downregulates CD73/adenosine signaling and promotes DC maturation in *vivo*. a** Representative bioluminescent imaging of mice bearing orthotopic EGFR-mutant LLC-19del lung tumors treated with saline (Ctrl) or osimertinib. **b** Tumor growth curves of orthotopic EGFR-mutant tumors following treatment. **c** Adenosine levels in the tumor microenvironment measured by adenosine assay kit. **d** Flow cytometry analysis of CD73 expression in tumor cells. **e** Representative flow cytometry plots of tumor-infiltrating DCs. **f** Quantification of DC abundance based on flow cytometry analysis. **g** Flow cytometry analysis of DC maturation markers.


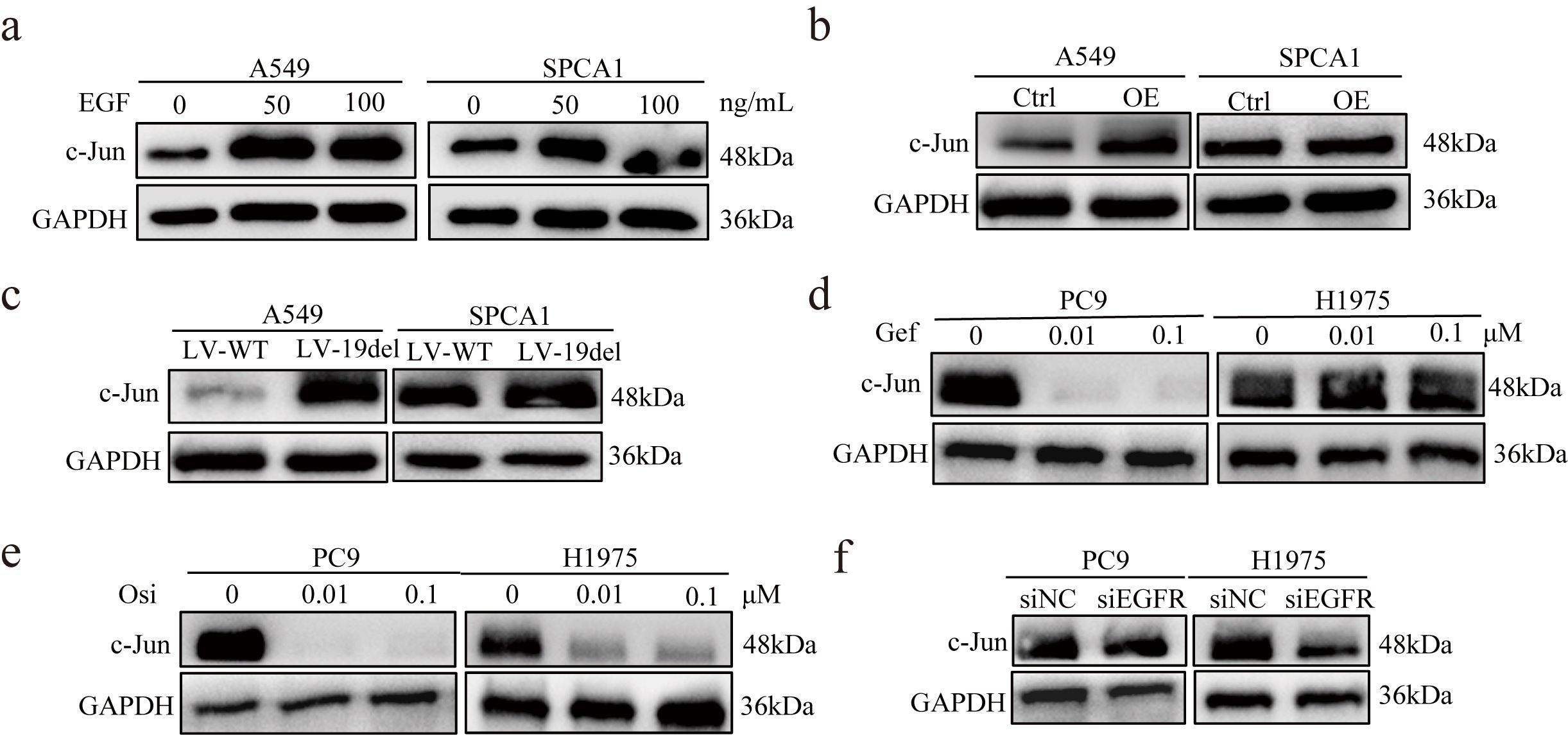


**Figure S9. c-Jun expression is regulated by EGFR activation. a** Immunoblot analysis of c-Jun expression in A549 and SPCA1 cells treated with different concentrations of EGF. **b** c-Jun expression in A549 and SPCA1 cells overexpressing EGFR plasmids, analyzed via immunoblotting. **c** Immunoblot analysis of c-Jun expression in A549 and SPCA1 cells transfected with EGFR 19del virus. **d** Immunoblot analysis of c-Jun expression in PC9 and H1975 cells treated with gefitinib at different concentrations. **e** c-Jun expression in PC9 and H1975 cells treated with osimertinib, analyzed via immunoblotting. **f** c-Jun expression in PC9 and H1975 cells after EGFR knockdown, analyzed using immunoblotting.


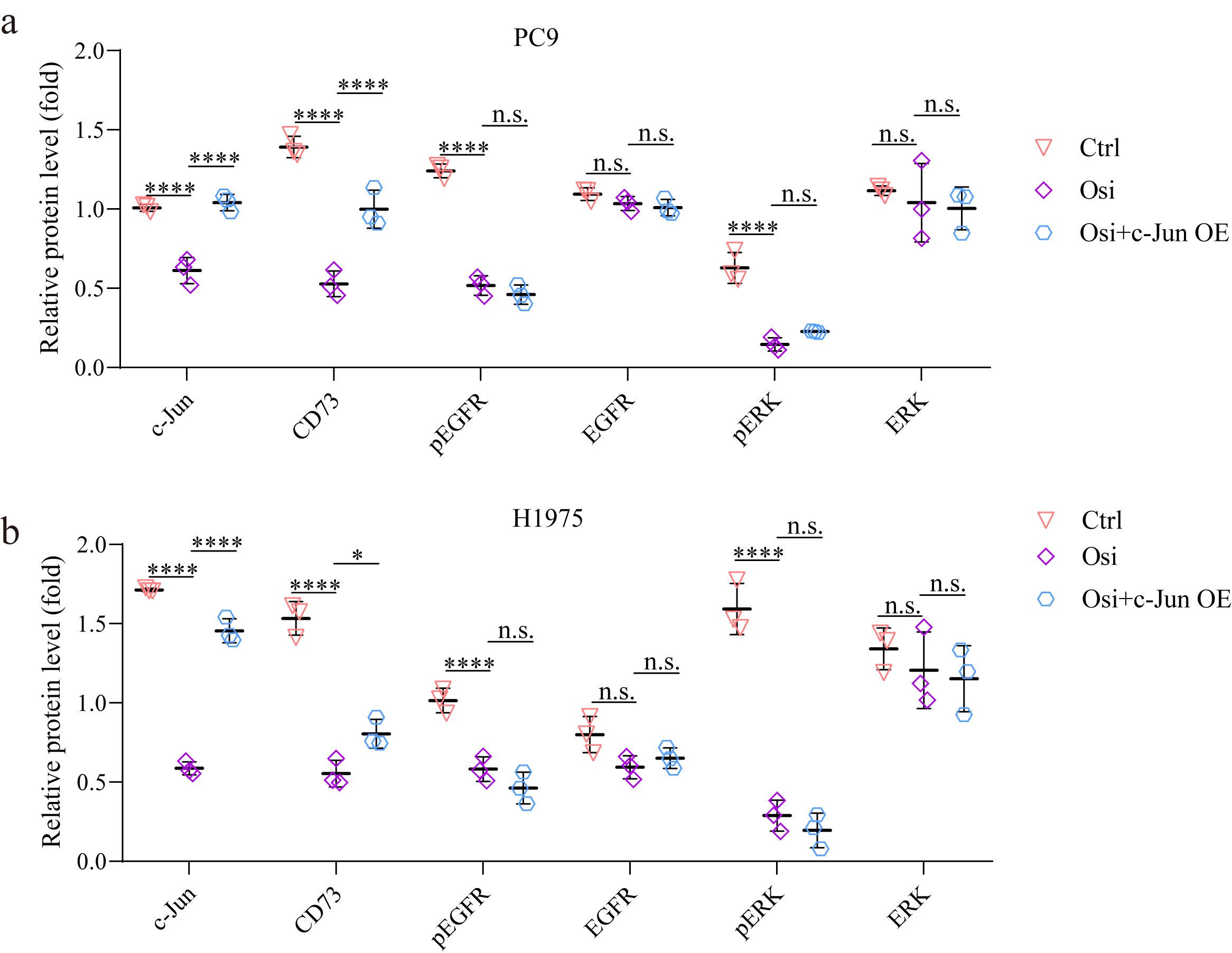


**Figure S10. Quantitative analysis of protein expression by Immunoblotting.** **a-b** Quantification of of EGFR, p-EGFR, ERK, p-ERK, c-Jun, and CD73 protein levels in PC9 and H1975 cells after treatment with osimertinib or osimertinib plus c-Jun overexpression. **P* < 0.05, *****P* < 0.0001, n.s., no significance.


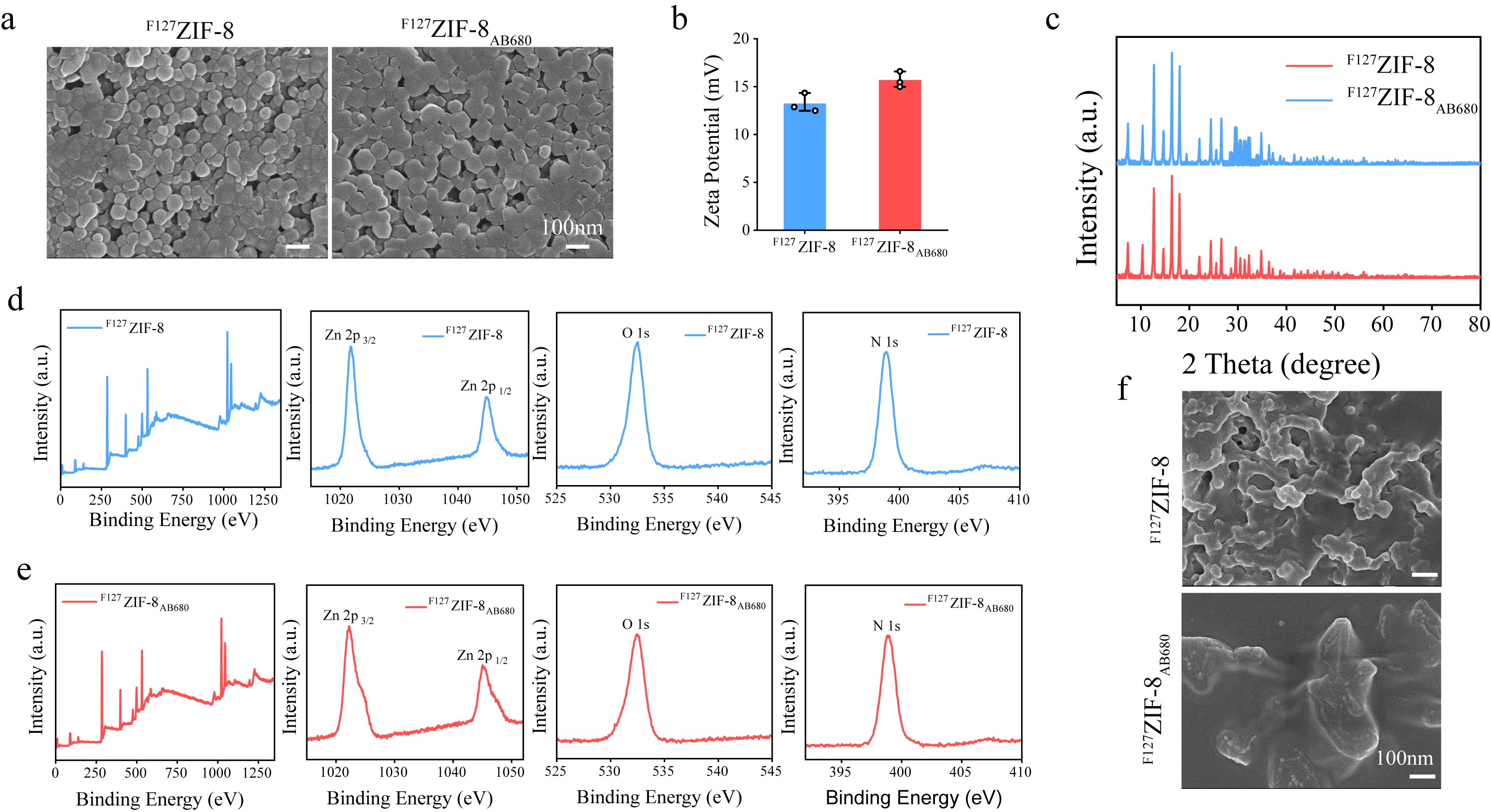


**Figure S11. Characteristic of NPs. a** Representative SEM image of ^F127^ZIF-8 and ^F127^ZIF-8_AB680_. Scale bar, 100nm. **b** Zeta potentials of ^F127^ZIF-8 and ^F127^ZIF-8_AB680_. **c** XRD pattern of ^F127^ZIF-8 and ^F127^ZIF-8_AB680_ NPs. **d-e** XPS spectra of ^F127^ZIF-8 and ^F127^ZIF-8_AB680_ NPs, XPS high-resolution scans of Zn 2p peaks, O 1s peaks and N 1s peaks in ^F127^ZIF-8 and ^F127^ZIF-8_AB680_ NPs. **f** SEM image of ^F127^ZIF-8 and ^F127^ZIF-8_AB680_ NPs treated with acidic PBS solution (pH = 6.0) for 24 h. Scale bar, 100nm.


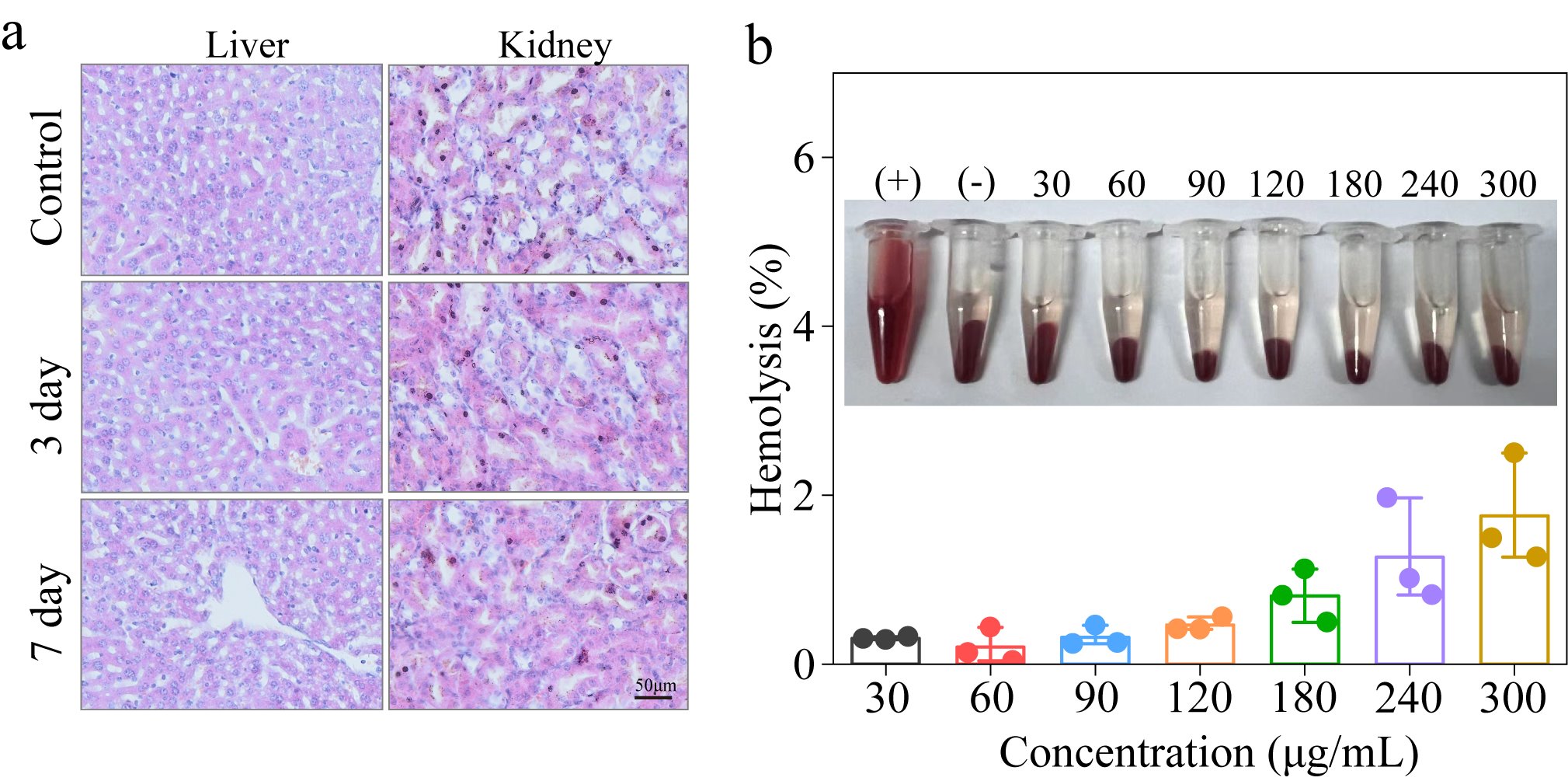


**Figure S12. Biosafety evaluation of ^F127^ZIF-8_AB680_. a** H&E-stained images of liver and kidney for 3 or 7 d of the administration of ^F127^ZIF-8_AB680_ NPs. **b** Hemolysis assay of ^F127^ZIF-8_AB680_, the embedded image is a picture of red blood cells after ^F127^ZIF-8_AB680_ treatment.


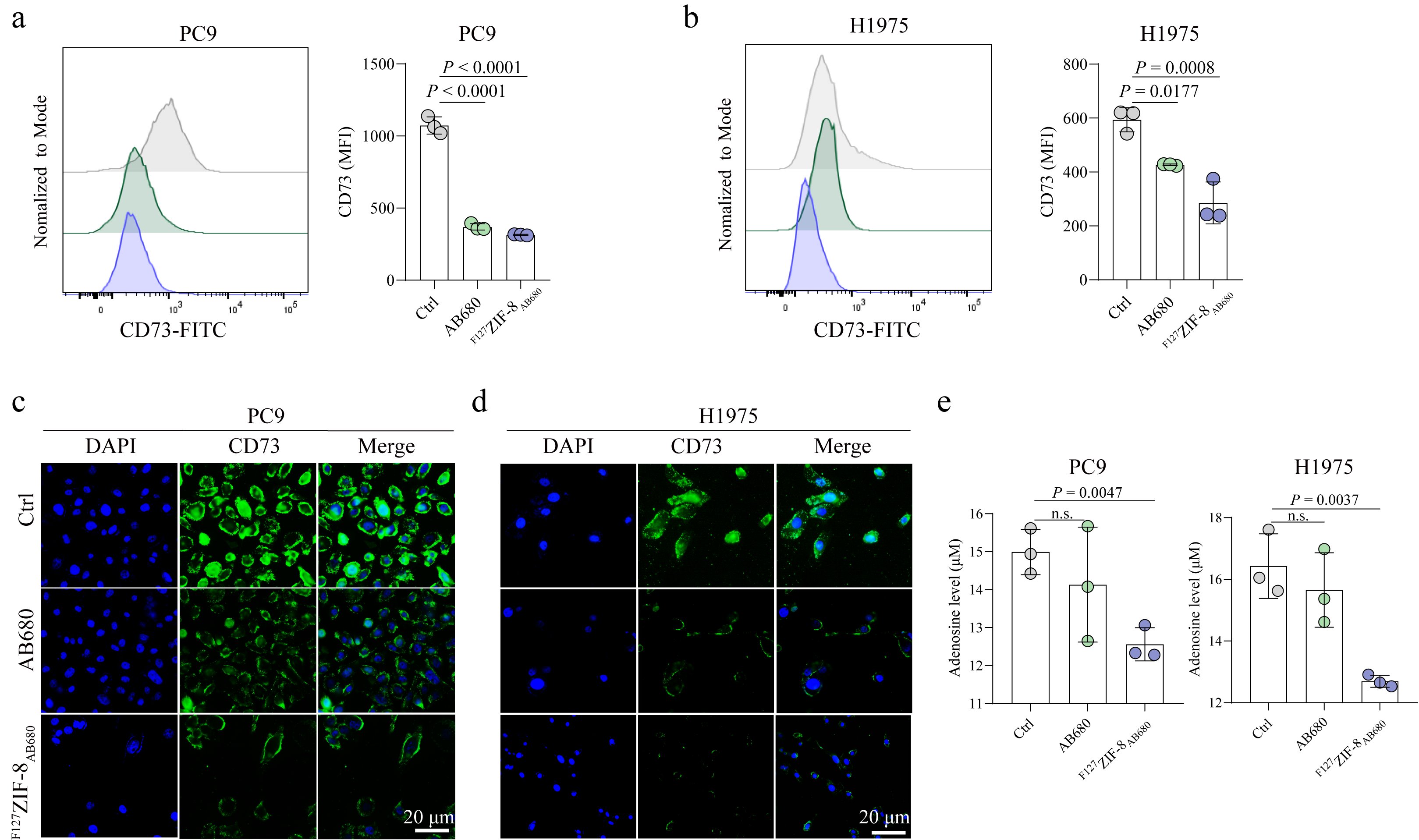


**Figure S13. Inhibition of CD73 expression and adenosine production in EGFR-mutant cells by AB680 and ^F127^ZIF-8_AB680_ in vitro. a** Flow cytometry analysis of CD73 expression in PC9 cells after 24-hour treatment with AB680 and ^F127^ZIF-8_AB680_ in vitro. **b** Flow cytometry analysis of CD73 expression in H1975 cells after 24-hour treatment with AB680 and ^F127^ZIF-8_AB680_ in vitro. **c** Confocal microscopy images of CD73 expression in PC9 cells treated for 24 hours with AB680 and ^F127^ZIF-8_AB680_ in vitro. **d** Confocal microscopy images showing CD73 expression in H1975 cells following 24-hour treatment with AB680 and ^F127^ZIF-8_AB680_ in vitro. **e** Measurement of adenosine levels in PC9 and H1975 cells treated with AB680 and ^F127^ZIF-8_AB680_ for 24 hours in vitro using an adenosine detection kit. n.s., no significnace.


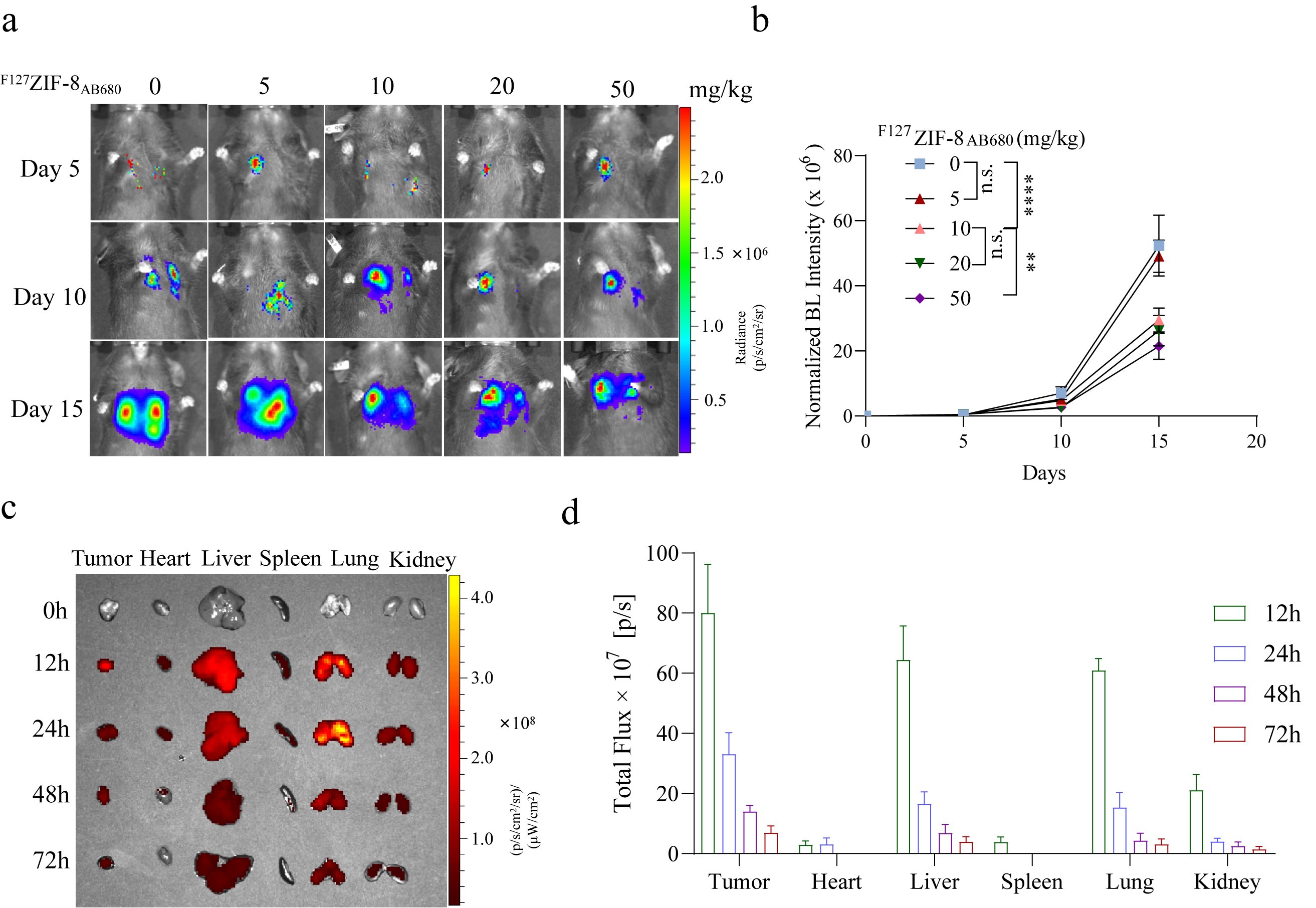


**Figure S14. Dose-dependency and biodistribution of ^F127^ZIF-8_AB680_ in orthotopic EGFR-mutant LLC tumors. a** Representative bioluminescent imaging of orthotopic LLC-19del-luciferase tumor-bearing mice treated with different doses of ^F127^ZIF-8_AB680_. **b** Tumor growth curves of mice receiving varying doses of ^F127^ZIF-8_AB680_. **c** Biodistribution of ^F127^ZIF-8_AB680_ in major organs (heart, liver, spleen, lung, kidney) and tumor tissue, assessed by in vivo pharmacokinetic analysis. **d** Quantification of ^F127^ZIF-8_AB680_ accumulation in tumors relative to major organs. ***P* < 0.01, *****P* < 0.0001, n.s., no significance.


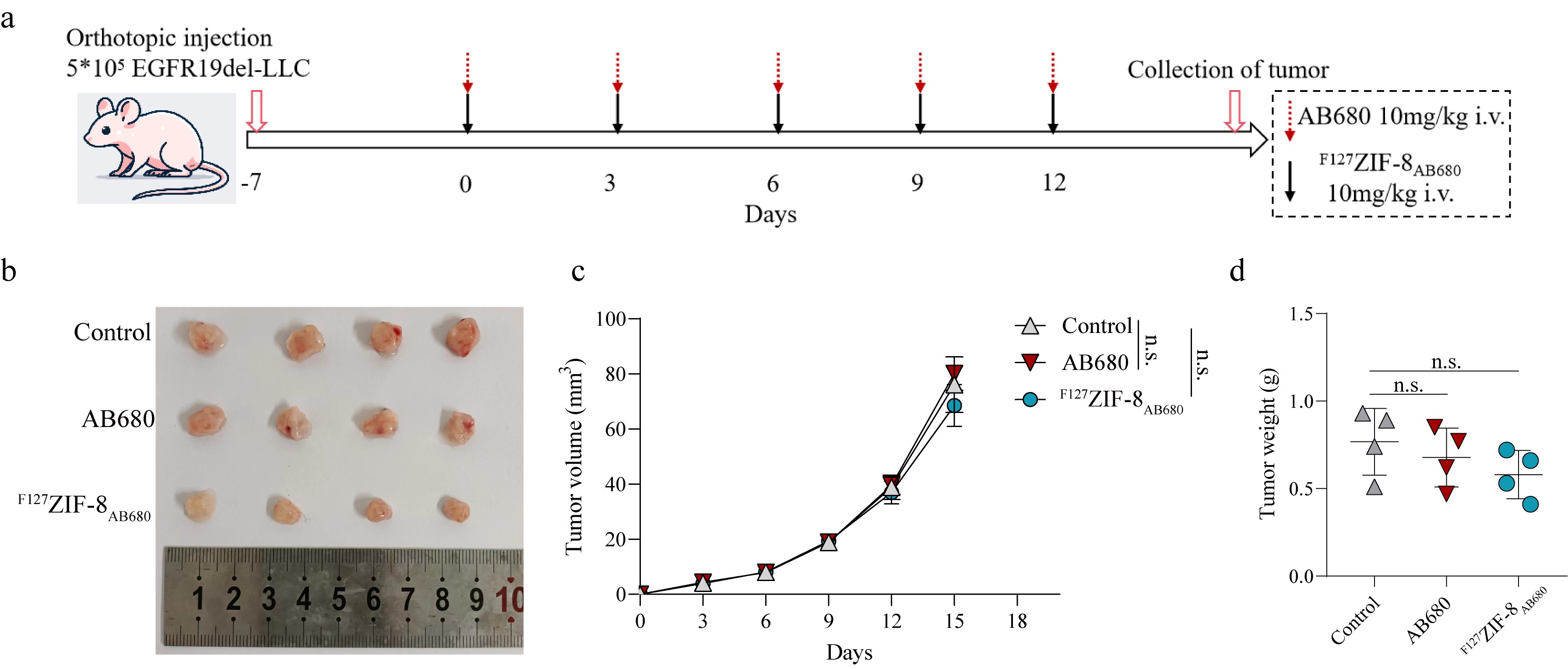


**Figure S15. AB680 and ^F127^ZIF-8_AB680_ has no anti-tumor effect in nude mice. a** Schematic showing the schedule of AB680 and ^F127^ZIF-8_AB680_ treatment in EGFR-19del-LLC subcutaneous model of nude mice. **b-d** Image (b), volumes (c) and weights (d) of the AB680- or ^F127^ZIF-8_AB680_-treated versus control EGFR-19del-LLC orthotopic tumors in nude mice (n = 4 samples per group). n.s., no significance.


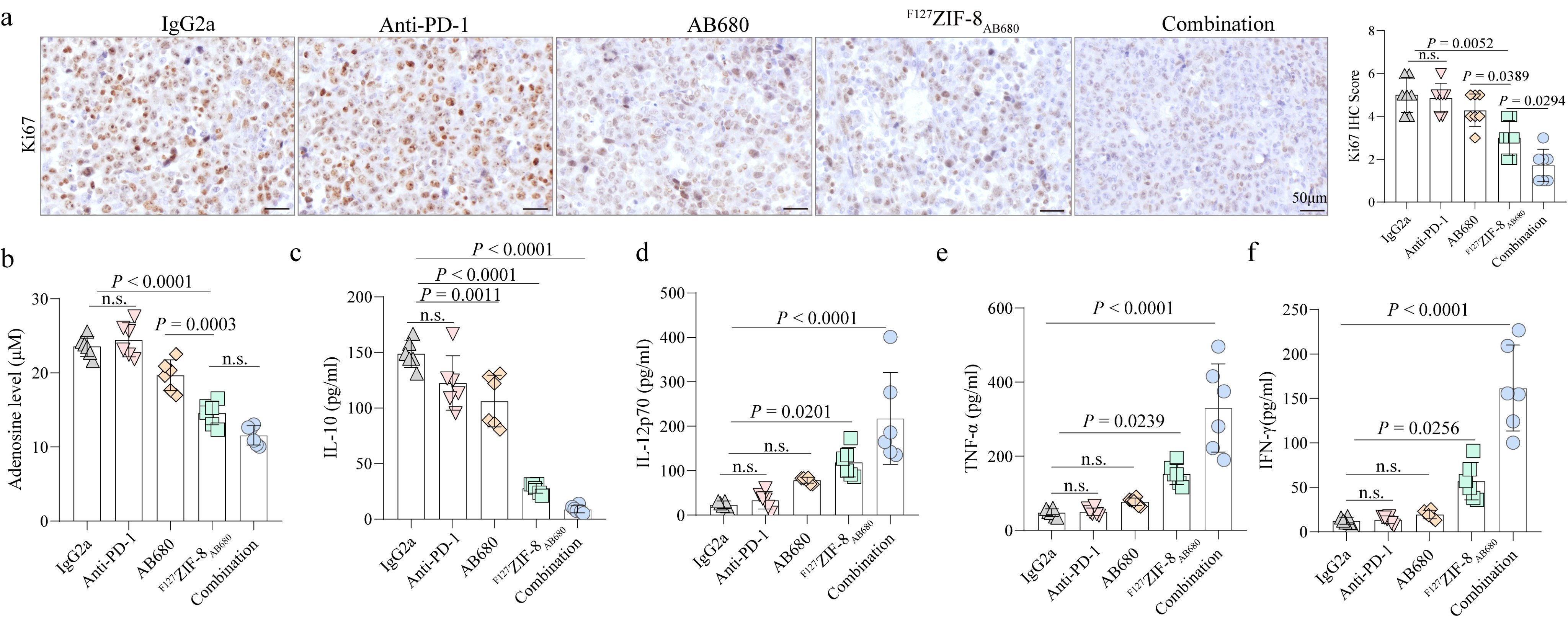


**Figure S16. Immunohistochemistry and cytokine profiling in tumor and serum samples from treated mice. a** Representative images and quantiﬁcation of immunohistochemistry staining of Ki-67 in the lung orthotopic model from the indicated treatment groups (n = 6 samples per group). Scale bar, 50 μm. **b** The level of adenosine in tumor tissue of each group at the endpoint (n = 6 samples per group). **c-f** The protein level of IL10 (c), IL12p70 (d), TNF-α (e) and IFN-γ (f) in mouse serums from the indicated treatment groups. (n = 6 samples per group), n.s, no significnace.


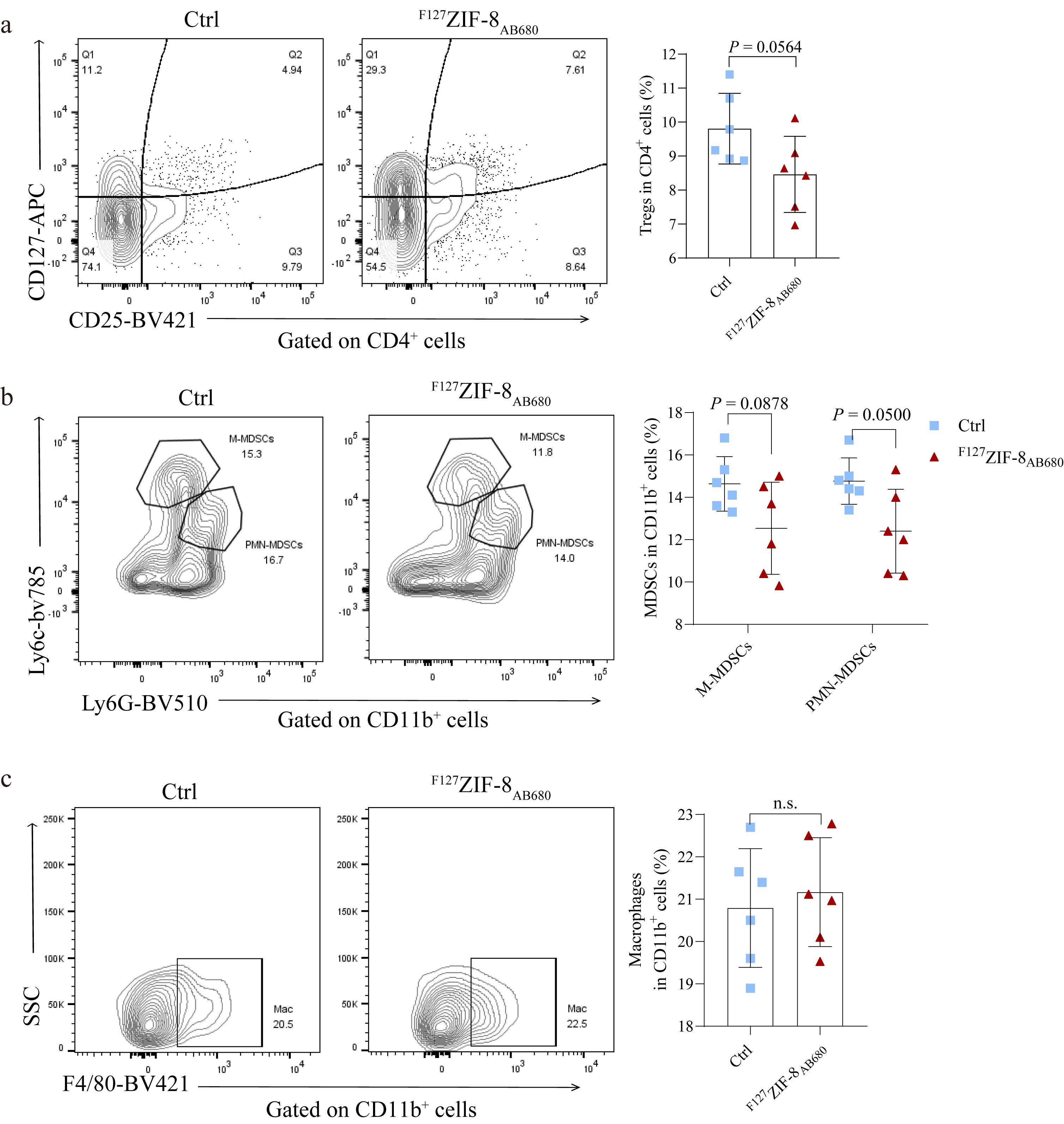


**Figure S17. Analysis of immunosuppressive cell populations in the tumor microenvironment after treatment.** Tumors from mice treated with the indicated regimens were analyzed by flow cytometry. **a** Frequency of regulatory T cells (Tregs, defined as CD25^high^CD127^low^ of CD4^+^ T cells). **b** Frequencies of monocytic MDSCs (M-MDSCs, defined as CD11b^+^Ly6G^-^Ly6C^high^) and polymorphonuclear MDSCs (PMN-MDSCs, defined as CD11b^+^Ly6G^+^Ly6C^low^). **c** Frequency of tumor-associated macrophages (defined as F4/80^+^ cells). n.s., no significance.


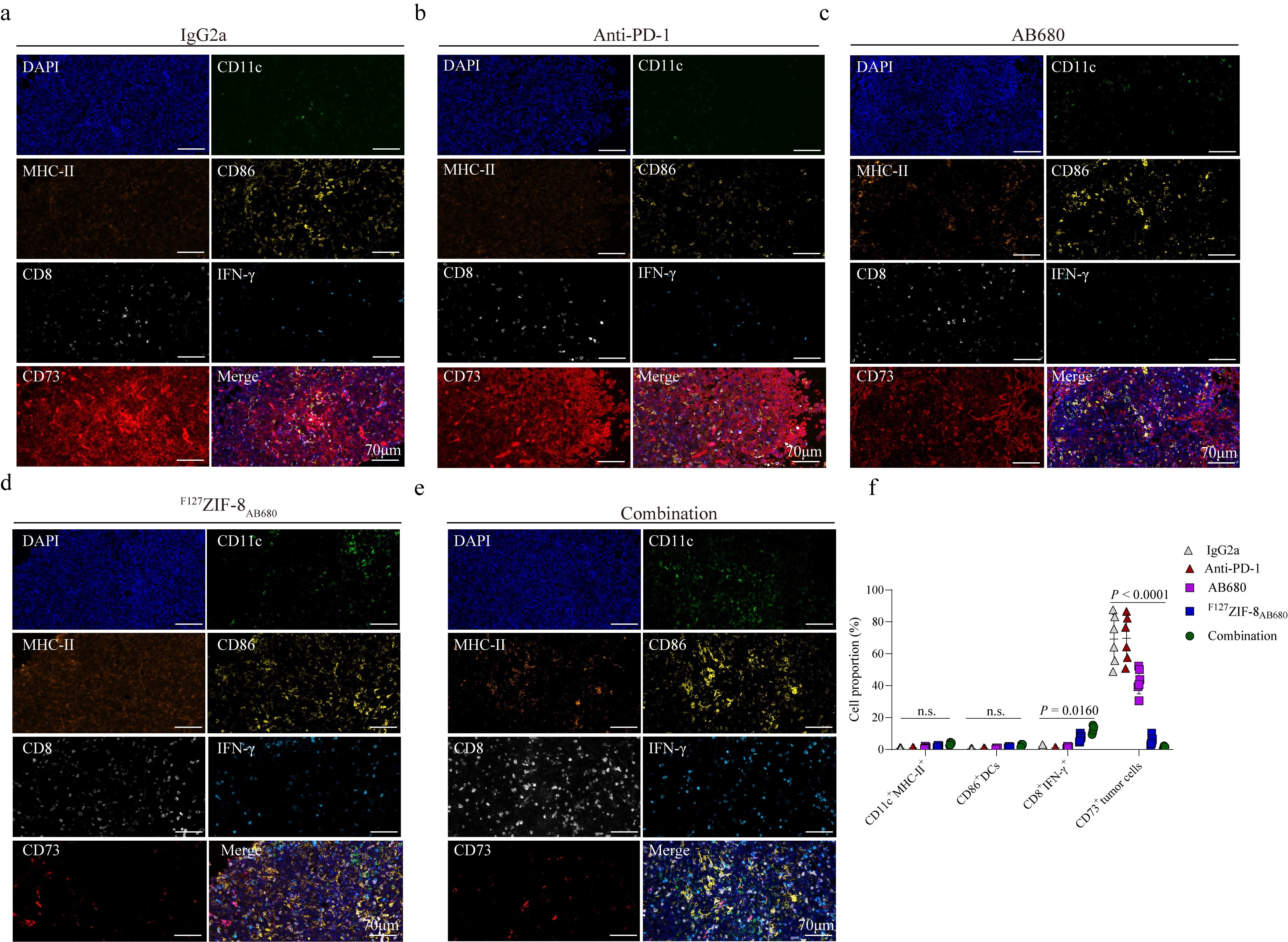


**Figure S18. The single-channel staining of the immune microenvironment in tumor tissues across different treatment groups.** Fluorescence signals for CD11c (in green), MHC-II (in orange), CD86 (in yellow), CD8 (in white), IFN-γ (in light blue) and CD73 (in red) are presented in tumor tissues across IgG2a treatment group (a), Anti-PD-1 treatment group (b), AB680 treatment group (c), ^F127^ZIF-8_AB680_ treatment group (d) and combination group (e). Scale bar, 70μm. n.s., no significance.

**Table S1.** All incorporated patients’ characteristics.

| **Characteristics** | **Qilu Hospital of Shandong University**  **(n = 20)** | **Shandong Cancer Hospital and Institute**  **(n = 52)** |
| --- | --- | --- |
| **Age** |  |  |
| < 65 | 14 (70.0%) | 38 (73.1%) |
| ≥ 65 | 6 (30.0%) | 14 (26.9%) |
| **Sex** |  |  |
| Female | 13 (65.0%) | 27 (51.9%) |
| Male | 7 (35.0%) | 25 (48.1%) |
| **Smoking history** |  |  |
| No | 12 (60.0%) | 39 (75.0%) |
| Yes | 8 (40.0%) | 13 (25.0%) |
| **Stage** |  |  |
| III | 2 (10.0%) | 5 (9.6%) |
| IV | 18 (90.0%) | 47 (90.4%) |
| **EGFR mutation type** |  |  |
| 19del | 7 (35.0%) | 22 (42.3%) |
| L858R | 4 (20.0%) | 13 (25.0%) |
| 18del | 0 (0%) | 1 (1.9%) |
| WT | 9 (45.0%) | 16 (30.8%) |

**Table S2.** List of antibodies and reactive dye used for flow cytometry.

| **Antibody** | **Reactivity** | **Color** | **Company** | **Cat No.** | **Clone** |
| --- | --- | --- | --- | --- | --- |
| CD3 | Human | PE | Biolegend | 300439 | UCTH1 |
| CD45 | Human | [Brilliant Violet 510™](https://www.bioec.cn/product/5f601cf2afef2b4993dd37f5) | Biolegend | 304035 | HI30 |
| CD8 | Human | FITC | Biolegend | 344704 | SK1 |
| CD8 | Human | [Brilliant Violet 510™](https://www.bioec.cn/product/5f601cf2afef2b4993dd37f5) | Biolegend | 344732 | SK1 |
| [Lineage (CD3/14/19/20/56)](https://www.bioec.cn/product/5f601ce5afef2b4993dcdee7) | Human | FITC | Biolegend | 348701 | UCHT1; HCD14; HIB19; 2H7; HCD56 |
| CD11c | Human | PE | Biolegend | 371503 | S-HCL-3 |
| CD14 | Human | FITC | Biolegend | 325603 | HCD14 |
| HLA-DR | Human | [Alexa Fluor® 700](https://www.bioec.cn/product/5f601ce2afef2b4993dccd23) | Biolegend | 327014 | LN3 |
| CD86 | Human | PerCP-Cyanine5.5 | Biolegend | 305419 | IT2.2 |
| CD80 | Human | [Brilliant Violet 421™](https://www.bioec.cn/product/5f601d00afef2b4993ddc968) | Biolegend | 305221 | 2D10 |
| Ki-67 | Human | PerCP-Cyanine5.5 | Biolegend | 350519 | Ki-67 |
| TNF-α | Human | [Alexa Fluor® 700](https://www.bioec.cn/product/5f601ce2afef2b4993dccd23) | Biolegend | 502927 | MAb11 |
| IFN-γ | Human | APC | Biolegend | 502511 | 4S.3B |
| CD73 | Human | FITC | Biolegend | 344015 | AD2 |
| EGFR (deleted E746 + L747 + R748 + E749 + A750) | Human |  | abcam | Ab227737 | SP111 |
| CD45 | Mouse | PE/Cyanine7 | Biolegend | 147704 | I3/2.3 |
| CD3 | Mouse | [Brilliant Violet 605™](https://www.bioec.cn/product/5f601cecafef2b4993dd064d) | Biolegend | 100237 | 17A2 |
| CD8a | Mouse | PerCP-Cyanine5.5 | eBioscience^TM^ | 45-0081-82 | 53-6.7 |
| CD4 | Mouse | FITC | Biolegend | 116003 | RM4-4 |
| CD4 | Mouse | PerCP-Cyanine5.5 | Biolegend | 116012 | RM4-4 |
| CD11c | Mouse | FITC | Biolegend | 117305 | N418 |
| IA/IE | Mouse | PerCP-Cyanine5.5 | Biolegend | 107625 | M5/114.15.2 |
| CD86 | Mouse | PE | Biolegend | 105007 | GL-1 |
| CD80 | Mouse | [Brilliant Violet 421™](https://www.bioec.cn/product/5f601d00afef2b4993ddc968) | Biolegend | 104732 | 16-10A1 |
| TNF-α | Mouse | PE | Biolegend | 506306 | MP6-XT22 |
| IFN-γ | Mouse | APC | Biolegend | 505809 | XMG1.2 |
| IFN-γ | Mouse | [Brilliant Violet 510™](https://www.bioec.cn/product/5f601cf2afef2b4993dd37f5) | Biolegend | 505841 | XMG1.2 |
| CD73 | Mouse | Alexa Fluor® 700 | Biolegend | 127229 | TY/11.8 |
| CD127 | Mouse | APC | Biolegend | S18006K | 158206 |
| CD25 | Mouse | [Brilliant Violet 421™](https://www.bioec.cn/product/5f601d00afef2b4993ddc968) | Biolegend | 101923 | 3C7 |
| Ly-6C | Mouse | [Brilliant Violet 785™](https://www.bioec.cn/product/5f601cf2afef2b4993dd37f5) | Biolegend | 128041 | HK1.4 |
| Ly-6G | Mouse | [Brilliant Violet 510™](https://www.bioec.cn/product/5f601cf2afef2b4993dd37f5) | Biolegend | 127633 | 1A8 |
| F4/80 | Mouse | eFluor™ 450 | Ebiosience | 48-4801-82 | BM8 |
| CD11b | Mouse/Human | FITC | Biolegend | 101206 | M1/70 |
| Purified anti-mouse CD16/32 | Mouse |  | Biolegend | 101301 |  |

**Table S3.** List of the specific primers used.

| **Gene** | **Sense** | **Antisense** |
| --- | --- | --- |
| Homo-CD73 | GAAAGTGAGGGGTGTGGACG | CCTTCCGCCCATCATCAGAA |
| Homo-EGFR | CGCCCATATCTGCTGCTCAAGAC | CTCTGTGCCTCCTTGTGCCTTTC |
| Homo-c-Jun | GAGGCAGGAGAATCGCTTGAACC | CTCTTGTTGCCCTGGTTGGAGTG |
| Homo-β-actin | TATGCTCTCCCTCACGCCATCC | GTCACGCACGATTTCCCTCTCAG |

**Table S4.** List of primary antibodies and secondary antibodies used for immunoblotting.

| **Antibodies** | **Apply** | **Source** | **Cat No.** |
| --- | --- | --- | --- |
| Anti-CD73 | 1:1000 | Abcam | Ab133582 |
| Anti-EGFR | 1:1000 | Bioss | Bsm-52317R |
| Anti-phospho-EGFR (Tyr1068) | 1:1000 | Abcam | Ab40815 |
| Anti-ERK1/2 | 1:1000 | CST | 4695 |
| Anti-phospho-ERK1/2 (Thr202/Tyr204) | 1:1000 | CST | 4370 |
| Anti-AKT | 1:1000 | CST | 4691 |
| Anti-phospho-AKT (Thr308) | 1:1000 | CST | 13038 |
| Anti-PKCδ | 1:1000 | CST | 9616 |
| Anti-phospho-PKCδ (Thr505) | 1:1000 | CST | 9374 |
| Anti-P65 | 1:1000 | Abcam | Ab16502 |
| Anti-phospho-P65 (S536) | 1:1000 | Abcam | Ab76302 |
| Anti-c-Jun | 1:1000 | CST | 9165 |
| GAPDH | 1:5000 | Proteintech | 60004-1-Ig |
| HRP-conjugated Goat Anti-Rabbit IgG (H+L) (Affinity Purified) | 1:10000 | zsbio | ZB-2301 |
| HRP-conjugated Goat Anti-Mouse IgG (H+L) (Affinity Purified) | 1:10000 | zsbio | ZB-2305 |

HRP: Horseradish Peroxidase

**Table S5.** List of antibodies used for immunohistochemistry (IHC) and multiplex immunofluorescent (mIF) staining.

| **Antibody** | **Company** | **Cat No.** | **Reactivity** | **Dilution ratio** |
| --- | --- | --- | --- | --- |
| CD8 | abcam | ab199016 | Human | 1:200 |
| CD8 | abcam | ab217344 | Mouse | 1:200 |
| MHC-II | abcam | ab55152 | Human | 1:100 |
| MHC-II | abcam | ab23990 | Mouse | 1:100 |
| CD11c | abcam | ab52632 | Human | 1:100 |
| CD11c | abcam | ab219799 | Mouse | 1:100 |
| CD86 | abcam | ab239075 | Human | 1:200 |
| CD86 | abcam | ab119857 | Mouse | 1:200 |
| IFN-γ | ThemoFisher | PMC4031 | Human | 1:50 |
| IFN-γ | ThemoFisher | PMC4034 | Mouse | 1:50 |
| CK | zmbio | ZM-0069 |  | 1:100 |
| CD73 | abcam | ab133582 | Human | 1:100 |
| CD73 | abcam | ab175396 | Mouse | 1:100 |

**Table S6.** The identified differential metabolites of Liquid chromatography-mass spectrometry (LC-MS) analysis.

| **Name** | **Log2FC** | **P.value** | **FDR** | **OPLSDA.VIP** |
| --- | --- | --- | --- | --- |
| Adenosine | 4.803603331 | 1.08491E-06 | 0.000573918 | 1.691057724 |
| 4-Aminobenzoic acid | 2.131182163 | 3.43362E-06 | 0.000778398 | 1.689942486 |
| 4-Hydroxyphenyllactate | 6.803996102 | 4.41436E-06 | 0.000778398 | 1.689598973 |
| Hypoxanthine | 6.761519947 | 6.19915E-06 | 0.000819838 | 1.689054584 |
| Guanosine | -2.248454633 | 1.29213E-05 | 0.001199712 | 1.687551723 |
| 1-Methyladenosine | 5.593153894 | 1.58752E-05 | 0.001199712 | 1.687004544 |
| 5'-Methylthioadenosine | 6.025599811 | 1.43857E-05 | 0.001199712 | 1.6872691 |
| 5'-Deoxyadenosine | 4.742919768 | 4.44405E-05 | 0.002612114 | 1.683283863 |
| Xanthosine | -3.168134613 | 5.39532E-05 | 0.002854123 | 1.682390327 |
| CMP | 4.823310588 | 7.12165E-05 | 0.003424865 | 1.680819589 |
| 4-Nitrophenol | 1.986761862 | 8.28933E-05 | 0.003654214 | 1.679938588 |
| N6-methyladenosine | 5.487985653 | 0.000108739 | 0.004424856 | 1.678089844 |
| Alpha-D-Ribose 5-phosphate | 3.742733909 | 0.0001176 | 0.004443595 | 1.677464847 |
| Isopentenyladenosine | 3.255659189 | 0.000135304 | 0.004771728 | 1.676394659 |
| GMP | 4.10335301 | 0.000279269 | 0.007775437 | 1.669370836 |
| Hexose Disaccharide Monophosphate Pool | 1.706216715 | 0.000418209 | 0.010056014 | 1.664211441 |
| Uridine 5-monophosphate | 2.4630239 | 0.000707492 | 0.016272316 | 1.655615864 |
| Carnitine-C3 | 2.314189823 | 0.001358907 | 0.023962062 | 1.641325638 |
| Inosine | 3.433098873 | 0.001563563 | 0.024707105 | 1.637509002 |
| Deoxycytidine monophosphate | 7.631815587 | 0.001901054 | 0.026644062 | 1.631855154 |
| Uridine | -1.771765757 | 0.002348624 | 0.028893538 | 1.624954992 |
| Carnitine-C12 | 1.658541803 | 0.004417107 | 0.045439709 | 1.59976741 |
